# Beyond the Leak: Analyzing the Real-World Exploitation of Stolen Credentials Using Honeypots

**DOI:** 10.3390/s25123676

**Published:** 2025-06-12

**Authors:** Matej Rabzelj, Urban Sedlar

**Affiliations:** Faculty of Electrical Engineering, University of Ljubljana, 1000 Ljubljana, Slovenia; urban.sedlar@fe.uni-lj.si

**Keywords:** cyberattack analysis, data breach, honeypots, leaked credentials, service modeling, username and password analysis

## Abstract

This study presents one of the most extensive analyses of the lifecycle of leaked authentication credentials to date, bridging the gap between database breaches and real-world cyberattacks. We analyze over 27 billion leaked credentials—nearly 4 billion unique—using a sophisticated data filtering and normalization pipeline to handle breach inconsistencies. Following this analysis, we deploy a distributed sensor network of 39 honeypots running 14 unique services across 9 networks over a one-year-long experiment, capturing one of the most comprehensive authentication datasets in the literature. We analyze leaked credentials, SSH and Telnet session data, and HTTP authentication requests for their composition, characteristics, attack patterns, and occurrence. We comparatively assess whether credentials from leaks surface in real-world attacks. We observe a significant overlap of honeypot logins with common password wordlists (e.g., Nmap, John) and defaultlists (e.g., Piata, Mirai), and limited overlaps between leaked credentials, logins, and dictionaries. We examine generative algorithms (e.g., keywalk patterns, hashcat rules), finding they are widely used by users but not attackers—unless included in wordlists. Our analyses uncover unseen passwords and methods likely designed to detect honeypots, highlighting an adversarial arms race. Our findings offer critical insights into password reuse, mutation, and attacker strategies, with implications for authentication security, attack detection, and digital forensics.

## 1. Introduction

The rapid growth in internet traffic has accelerated the digitization of numerous services and heightened the risks associated with online data breaches. As businesses and individuals increasingly rely on digital platforms for everyday activities, sensitive personal and corporate information, along with critical authentication credentials, are frequently transmitted and stored online. This situation provides a fertile ground for adversaries to exploit security vulnerabilities and orchestrate cyberattacks, often resulting in the theft of digital identities and other sensitive data.

Especially valuable are stolen digital identities obtained in cyberattacks known as database breaches [1]. Stolen data are frequently sold to the highest bidders on the dark web, but oftentimes also leak into the public part of the internet as the novelty of the attack slowly wears off [2]. Nevertheless, the implications of compromised credentials extend far beyond the initial account takeover attack [3]. Leaked credentials serve as a stepping-stone for further attacks, as victims of the crime often notice the leaks when it is already too late [4]. The resulting cyberattacks range from straightforward public disclosures of confidential information to even some of the most sophisticated attacks directly impacting the critical infrastructure and sovereignty of global superpowers [5].

To counteract these evolving threats, cybersecurity researchers employ various defensive strategies, one of the most effective being the use of honeypots. Either as a proactive security mechanism or a research tool, honeypots play a pivotal role in understanding and mitigating cyberthreats. They are designed to masquerade as part of the network’s active resources, deliberately configured to be susceptible to attacks. By mimicking real production environments (from simple service facades to complex, high-interaction systems), honeypots attract attackers, divert them from critical assets, and capture detailed information about their tactics, techniques, and procedures [6]. These systems can emulate various protocols and device types, including Information Technology (IT) networks, Internet of Things (IoT) devices, and industrial control systems, enabling broad and realistic attack surface modeling [7]. By analyzing attackers’ interactions with honeypots, researchers can gain insights into attack patterns, identify emerging threats, and better understand the behavior of malicious actors in the cyberthreat landscape.

This paper presents a multifaceted investigation into the lifecycle, usage, exploitation, and broader context of authentication credentials. It is grounded in the exploration of massive leaked credentials datasets and rich cyberattack data captured across a distributed network of honeypots and further supported by comparative analyses involving wordlists and synthetically generated passwords. The paper unifies these four major datasets and integrates multiple lines of investigation—each substantial in its own right—into a cohesive study. The examination and cross-comparison of these large-scale, recent, real-world datasets form the core contribution of this work. Additionally, we introduce a scalable data ingestion, filtering, and normalization pipeline capable of handling heterogeneous leak sources and real-world attack traffic. To the best of our knowledge, this represents the most extensive empirical study of credential leakage and misuse to date.

Our investigation is motivated by several interrelated research questions. Our primary aim is to understand how credentials are created, leaked, reused, and exploited—and how attacker behavior aligns with available password resources. We begin by examining the structural characteristics and patterns of leaked credentials in the wild, focusing on how frequently usernames and passwords repeat or mutate. We then explore how these credentials are later exploited in real-world cyberattacks across different services and sectors, aiming to trace the transition from leakage to active abuse. In parallel, we assess the extent to which publicly available wordlists and dictionaries align with actual credentials observed in both leaks and attacks, shedding light on their practical relevance and limitations. We also investigate whether synthetically generated passwords or rule-based transformations can effectively replicate or predict credentials chosen by users or attackers. Finally, we ask how combining insights from all these datasets can support the development of stronger password security practices and inform better detection and defense mechanisms.

**Figure 1:**
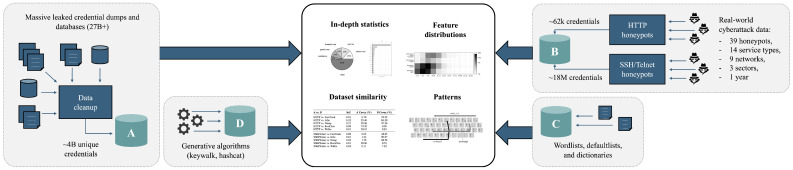
Graphical abstract displaying the scope of the work presented in this article. Four diverse datasets were collected, analyzed, and cross-compared: (**A**) a massive dataset of leaked credentials containing over 27B records that has been normalized and filtered using a sophisticated preprocessing pipeline; (**B**) a large dataset of nearly 18M records obtained from 39 globally deployed SSH, Telnet, and HTTP honeypots over a 1-year period; (**C**) a collection of wordlists and password dictionaries designed for brute-force attacks; and (**D**) generative algorithms, such as keywalks/hashcat. The analyses were first performed on each dataset individually and then comparatively to determine similarities and gain further insight into the role of password leaks in real-world hacking attempts.

The structure of this paper follows the chronological and methodological flow of our experiment. The ordering of our work is deliberate: leak data collection and analysis precede the honeypot deployment to support causal inference and allow for targeted and integrative insights. This sequencing allows us to study how leaked credentials might drive real-world attacks rather than treating these datasets as unrelated or concurrent observations. To ensure clarity and guide the reader throughout the course of the experiment, we emphasize that the work is structured around four datasets, each first examined individually and later combined in a final cross-analysis. A graphical overview of this structure is provided in Figure 1, which visualizes our approach: four independent datasets and a culminating analysis.

In Section 1, we introduce the rising concerns of data leakage incidents and discuss using honeypots to harvest malicious login attempts and aid cybersecurity research. In Section 2, we overview related work and the most common (mis)uses of the compromised data, and we outline key gaps in existing research. In Section 3, we concisely describe all four acquired datasets—massive credential leaks, honeypot-captured cyberattacks, wordlists including defaultlists and language dictionaries, and synthetic passwords resembling keyboard-walking patterns. We overview the honeypot deployments and system architecture, and we propose a scalable data ingestion, filtering, and normalization pipeline tailored to these data. In Section 4, we demonstrate the developed pipeline to process and analyze nearly 28 billion records of a highly inconsistent and heterogeneous leaked dataset. We reduce the ingested data to nearly 4 billion unique leaked records and set the methodological foundation for subsequent analyses, including investigating username and password reuse, overviewing impacted services, applications, and account domains, as well as reviewing credentials composition, characteristics, and patterns. Following the acquisition and analysis of the leaked data, we deploy a large-scale global honeynet to observe how such credentials may be used in practice. We analyze the honeypot datasets in two parts. In Section 5, we process over 750,000 captured HyperText Transfer Protocol (HTTP) requests, amounting to more than 70,000 logins. In Section 6, we analyze nearly 90 million Secure SHell (SSH) and Telnet events, resulting in 18 million username and password authentication attempts. We analyze broader contextual attack data, including attacker behavior, source distribution, and request, session, and client (meta)data alongside the login attempts, enabling a better understanding of adversarial credential selection strategies and their operational logic. In Section 7, we perform a cross-analysis of all the datasets. We compare leaked credentials with honeypot login attempts, measure overlap with wordlists, well-known default accounts, and dictionaries, and estimate dictionary coverage of both leaked and attacker-used passwords. We combine insights to reveal deeper behavioral patterns and estimate the overlap between known weak credentials and those actually used in attacks. We search for the presence of personal information in the datasets, hypothesize and verify the credential composition similarity between the sectors and services, and explore mutation rules for password prediction based on real-world data. Finally, in Section 8, we summarize our findings, conclude our work, and outline our prospects for future work.

## 2. Motivation and Related Work

### 2.1. Motivation

Identity leaks have become a widespread cybersecurity concern, driven by a rise in cyberattacks and the vast volume of leaked data now publicly available. High-profile breaches across organizations of all sizes, including Fortune 500 companies, are increasingly reported even in mainstream media [8]. A newer ransomware-based threat model further amplifies risk by exfiltrating data prior to encryption and demanding ransom for both decryption and non-disclosure. These trends highlight the urgent need for data-driven research into the real-world exploitation of leaked credentials and the development of effective defensive strategies.

### 2.2. Identity Leak Lookup Services

The rise in public breach disclosures has led to the development of identity leak lookup services that notify users of exposed data and allow email or domain-based queries. Notable platforms include Have I Been Pwned? (HIBP), Dehashed, IntelX.io, and SpyCloud, which vary by database size, freshness, and feature set. Typically, these tools compete in terms of how vast and how recent their databases are, but many have seen their rise and demise due to a substantial amount of manual work required to keep them updated. Some, like HIBP, employ privacy-preserving lookup methods based on k-anonymity and are used by vendors and Computer Emergency Response Teams worldwide [9]. In parallel, tech giants such as Microsoft, Apple, and Google have integrated similar breach-checking tools into their ecosystems. For example, Apple states their password monitoring feature matches the user’s Password AutoFill keychain against a continuously updated and curated list of 1.5 billion passwords known to have been exposed in leaks from different online organizations [10].

### 2.3. Leak Sources, Quantity, and Quality

Researchers have conducted studies on leak sources, quantity, and quality [11,12,13,14]. These works reported a dramatic rise in publicly available leak data from 800 million accounts in 2013 [11] to over 26 billion records by 2024 [8]. This explosion has been driven in part by the emergence of merged and repackaged leak bundles, such as BreachCompilation, COMB21, and Collection #1-5, which are often redistributed via breach forums, file-sharing platforms, or Telegram channels [2]. These compilations typically mix old and new data, exacerbating redundancy and reducing overall quality. More recent leaks like the Naz.API dataset, likely derived from credential-stealing malware (e.g., RedLine, LokiBot, Lumma), also include a service login Uniform Resource Locator (URL) tied to specific websites and mobile applications. Researchers have also focused on identifying leak distribution methods [2,11] and automating their discovery and parsing [12,15]. Despite these efforts, leak sources remain highly dispersed, often short-lived, and increasingly hosted on ephemeral platforms, complicating reliable and timely detection. Beyond technical issues, a more profound problem seems to be rooted in bad password hygiene and significant rates of password reuse [16]. This is especially concerning as the amount of traffic grows, as passwords still remain the predominant authentication method despite the industry push for a transition towards multi-factor, token, and key-based authentication schemes [17].

### 2.4. Analysis and Weaponization of Leaked Credentials

Prior work has analyzed leaked password datasets to assess user behavior, credential security, and the potential for exploitation. Early research [14] examined nearly 1 billion leaked email–password pairs to identify weak hashing schemes, frequent reuse, and predictable evolution patterns. Later, researchers [18] analyzed password composition for the dataset of the same size and reported on the use of keyboard patterns. Several studies have examined how leaked credentials are weaponized for identity theft and malicious activities. Thomas et al. [19] analyzed phishing kits and keyloggers, finding that phishing yielded much higher exposure rates compared to data breaches for valid Google account passwords. Other work, such as [20], demonstrated how publicly leaked datasets can be de-anonymized by linking identifiers, revealing users’ hidden profiles. Onaolapo et al. [21] analyzed how criminals interact with leaked credentials by creating Gmail honeyaccounts and leaking them across the web to monitor attacker behavior. Most actors merely tested logins, but some attempted data theft, spam propagation, or full account takeovers. Leaked datasets have also fueled advances in password guessing. While brute-force and rule-based tools like HashCat and John the Ripper remain standard, recent advances such as GNPassGAN apply generative adversarial networks to generate more effective guesses, improving success rates over older methods [22].

### 2.5. Honeypots and Detection of Leaked Data

Existing research describes using honeypots to capture cyberattack data and analyze the supplied authentication credentials. Fraunholz et al. [23] deployed medium-interaction SSH honeypots using default credentials and recorded over 150,000 attacks in 106 days. They reported detecting occurrences of Mirai and Hajime botnets exploiting weak default credentials of IoT devices but found no correlation between the attacking Internet Protocol (IP) addresses and Tor exit nodes, free proxies, or known VPN servers. In another paper [24], the authors produced statistics of common username and password combinations used in attacks on honeypots. Their experiment consisted of up to three deployed honeypots, one of which was linked to a domain, and it lasted for 111 days. They recorded 302,164 login attempts from 2219 unique IP addresses and identified common dictionaries used for wordlist-based attacks. Pahal and Priya [25] deployed a traffic-logging honeypot for 10 days and captured SSH login credentials and IP metadata of originating requests, whereas other authors [26] described using much larger global honeypot networks to detect protocol-level attacks, but they did not focus on login credentials or evaluate the links between the attacks and leaked data.

### 2.6. Identified Gaps

Prior studies have typically examined either leaked credentials or honeypot data in isolation. Analyses of leaked data often rely on limited datasets, while honeypot-based studies have been constrained by small deployments and short observation periods. To address these gaps, we adopted a comprehensive, six-fold approach. First, we acquired and processed one of the largest known datasets of leaked credentials, spanning billions of entries across multiple years, obtained from numerous sources. Second, we deployed a large and diverse honeynet based on our prior development—including SSH, Telnet, and HTTP honeypots capable of emulating IoT devices, Linux servers, Application Programming Interfaces (APIs), and web applications—with extensive authentication monitoring and long-term global exposure. Third, we collected and analyzed widely used wordlists, default credential sets, and language dictionaries. Fourth, we generated synthetic passwords resembling keyboard patterns for comparison. Fifth, we first analyzed leak and honeypot datasets independently, examining their structure, quantity, quality, and attack signatures. And finally, sixth, we performed cross-analysis across all sources, enabling novel insights into real-world credential reuse and attack behavior at an unprecedented scale. To our knowledge, no previous study has undertaken such an integrated and large-scale analysis.

## 3. Data and Methodology

### 3.1. Leak Data and Processing

**Figure 2:**
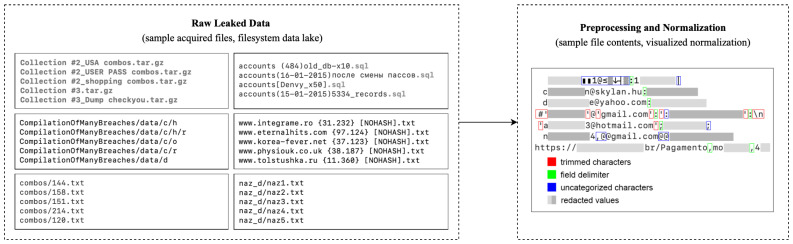
Depiction of file format variety, nomenclature, and organizational practices of breached databases. Partially redacted raw file contents reveal attempts at quasi-structured data, albeit with significant noise. Colored frames depict the delimiter detection and filtering approach.

We acquired over a terabyte of publicly available leak data from various sources, including large compilations, repackaged bundles, and standalone breach dumps dating up to 2023. The dataset comprised nearly 28 billion rows across more than 100,000 files, showing substantial variability in structure, format, and quality (Figure 2). Formats ranged from loosely structured comma-separated values (with inconsistent delimiters, illegal characters, and irregular quoting, etc.) to SQL dumps containing credentials and (un)related metadata. Most usable content consisted of raw text records with usernames, email addresses, or phone numbers paired with plaintext passwords. Filenames ranged from descriptive (e.g., breach source, region, or password type) to cryptic or non-informative labels.

**Figure 3:**
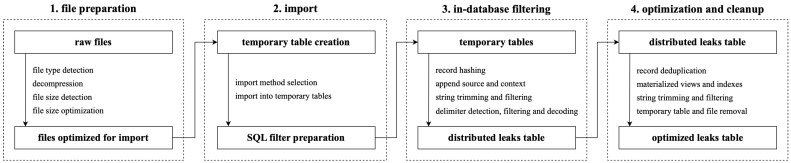
Visualization of the leak importing and processing sequence. Leaks were obtained in various file formats, preprocessed, and imported into temporary leak tables. In the database, SQL filters were prepared for each table to extract and sanitize leaked credentials. Lastly, all data was merged into a main distributed leaks table, deduplicated, and indexed.

To address this, we built a scalable pipeline for data sanitization, filtering, and normalization using the columnar Structured Query Language (SQL) database ClickHouse and an extract–load–transform workflow. The import script detected file types, decompressed archives, sorted contents, and extracted metadata (e.g., leak origin, service, region) while minimizing external processing and leveraging columnar compression. SQL-based filters performed delimiter detection using character frequency analysis, row hashing, metadata tagging, and field sanitization. We normalized email formats, trimmed non-alphanumeric wrappers, and parsed domains into second-level domains (SLDs) and top-level domains (TLDs). Resulting values (record hashes, leak sources, raw netlocs, extracted email addresses, usernames, passwords, SLDs, TLDs, service URLs, service paths, service query parameters, service SLDs, and service TLDs) were inserted into a distributed table, sorted by key cardinality to optimize compression and performance. Materialized views accelerated frequent queries, and the table was deduplicated by selected columns (Figure 3).

### 3.2. Honeypot Data and Services

We deployed 39 honeypot virtual machines, simulating 14 services and devices across 9 networks in 7 autonomous systems (AS). Based on our CyberLab platform [6,7], the honeypots covered HTTP, SSH, and Telnet protocols, offering username and password authentication and logging all request data. Deployments included both high- and low-interaction setups, ranging from fully interactive Linux systems to emulated web applications and APIs. Four honeypots were available through subdomains of distinct domain names, and one was embedded with hidden references to breached-domain identifiers. The honeypots were deployed in public cloud, academic research, and critical infrastructure networks across the globe and operated continuously from March 2023 to March 2024. They hosted logical combinations of services on multiple protocols and collected detailed server-side and client-side attack data. The deployed honeypot configurations are listed in Table 1.

**Figure 4:**
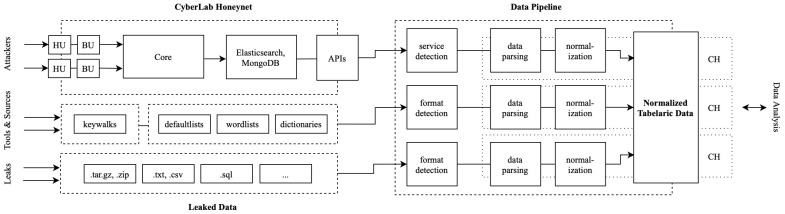
High-level architectural overview of the experiment depicting data sources, pipeline, and system components. The left side of the diagram depicts data sources and formats. Nodes denoted HU depict the distributed honeypot head units. Each head unit accepts attackers’ connections and tunnels the traffic into the backend units of the honeypots (BU), providing various degrees of interactivity. Backend units communicate with the core system to serve responses and capture attack data. Data are ingested into the data pipeline. Normalized data are stored in a distributed table inside a sharded database spanning across three nodes in a cluster.

SSH and Telnet honeypots accounted for most instances, capturing detailed authentication attempts and post-login session data, including commands, payloads, keystrokes, and rich client metadata such as HASSH fingerprints, client versions, host identifiers, and cryptographic negotiation parameters. HTTP honeypots simulated both stateful and stateless services depending on their interaction level, logging request payloads, headers, authentication data, and IP addresses. They also embedded client-side fingerprinting scripts and steganographic response encoding to enhance detection and attribution capabilities [7]. Authentication policies were tailored per deployment: some honeypots accepted predefined credentials, while others allowed login upon the n-th repeated attempt of a username and password combination. A high-level architectural overview of the experiment setup, including honeypot units and data collection, is depicted in Figure 4.

This article focuses on the captured authentication requests; however, we also incorporate broader attack data to contextualize login behavior, uncover attack patterns, and facilitate potential explanations of the observed activity and credential sets.

### 3.3. Wordlist and Dictionary Data

We collected and analyzed several widely used password wordlists, language dictionaries, and default credential sets to enable the evaluation of their prevalence in honeypot-captured attacks and leaked datasets. We selected five representative wordlists, often found preinstalled on penetration testing distributions such as Kali Linux, ranging from compact sets used in penetration testing (FastTrack, John the Ripper, Nmap) to larger (breach-derived) datasets (RockYou, Wifite). These lists varied in size (from a few hundred to over 14 million entries) and provided insight into different attacker profiles and guessing strategies.

Based on service URLs and email domains in leaked data, the geolocations of our honeypot deployments, and the country origins of observed attacks, we selected English, Russian, and German as the most relevant language groups and incorporated their dictionaries to evaluate the linguistic characteristics of captured credentials. We used the Online Plain Text English Dictionary (OPTED) and WordNet datasets, which are English lexical resources based on Webster’s 1913 Unabridged Dictionary and grouped cognitive synonyms, respectively, along with Wiktionary, a multilingual collaborative dictionary maintained by the Wikimedia Foundation.

Finally, to identify the use of default credentials, we compiled several username and password pair lists obtained from various sources, including by parsing strings from the Nessus Default Unix Accounts plugin family, and downloading dpl4hydra, open-sez.me, and Metasploit’s Mirai, Piata SSH, and Routers default lists. We preprocessed and deduplicated all lists and dictionaries where necessary.

### 3.4. Synthetic Password Data

We used a dedicated tool hashcat/kwprocessor to generate keyboard-based password patterns and enable analysis of keyboard-layout influence on password selection among the leaked data and honeypot-captured attacks. We generated keywalk pattern sets for the top five keyboard layouts inferred from generic and country code-based top-level domains of leaked data. We limited the generation of password patterns from 2 to 10 characters in length, used a full supported range of base characters, and defined a maximum of two keywalking direction changes on the keyboard. We also generated keywalk patterns for the Dvorak keyboard layout.

## 4. Leaked Credentials Analysis

### 4.1. Overview of Unique Data Records

**Figure 5:**
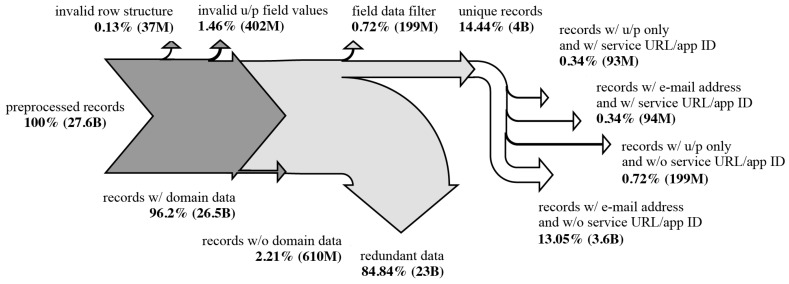
Visualization of leak importing and processing sequence of actual data. Arrows pointing up and down depict filtered and discarded data. Arrows pointing right indicate resulting data. Dark gray diagram component depicts import filtering; light gray, deduplication filtering; and the white component displays the resulting unique records.

We imported over 27 billion leaked records in total and sanitized and filtered the data as described in Section 3.1. More than 26 billion of the imported records contained usernames in the form of email addresses. Roughly 400 million records exhibited invalid or empty username or password strings in the credentials pair and were discarded. Deduplication by identity, password, and service URL (where available) combination yielded nearly 4 billion distinct records. In other words, the acquired data from some of the largest known leak collections, combinations, and breached databases exhibited a nearly seven-time redundancy in unique combinations of credential pairs, domains, and service URLs. An overview of the imported leaked data is given in Table 2. Data flow is visualized using the Sankey diagram in Figure 5. The counts of the resulting unique data are presented in Table 3.

### 4.2. Domain Data and Bundle Identifiers

**Figure 6:**
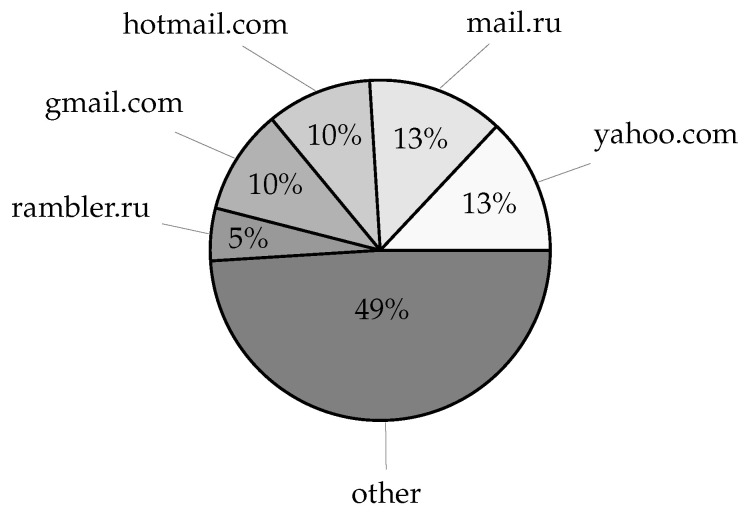
The most common domains of email addresses found in the leaked datasets. This figure reflects the most frequently used domain as login identifiers—though not necessarily the services that were breached. The prominence of domains suggests a mix of older breaches and regionally popular providers. The strong presence of Russian domains informed the inclusion of Russian in our linguistic password analysis. These distributions offer valuable context for profiling the geographical and demographic scope of the leaks.

**Figure 7:**
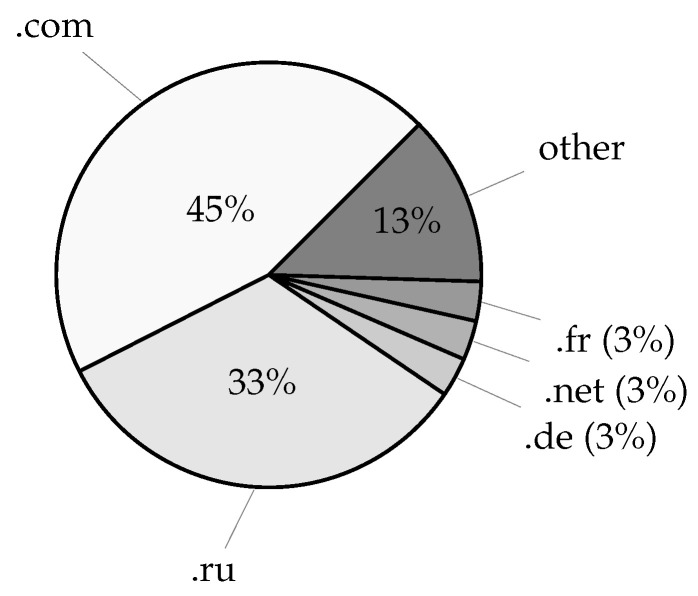
The most common TLDs extracted from email addresses in the leaked datasets. The dominance of .com (45%) reflects the global use of commercial service providers, while the strong showing of .ru (33%) highlights the significant portion of breaches involving Russian accounts. Other notable TLDs include .de, .net, and .fr, indicating German and French-speaking user populations. The TLD distribution supports regional profiling of leaks and informs downstream comparative analyses, such as the selection of representative language dictionaries and keyboard layouts for password composition studies.

The leaked data included over 27 million distinct domain names from credentials and over 4 million distinct domain names from service login URL addresses, excluding subdomain variations. Given the latest Domain Name Industry Brief Quarterly Report [27], this amounts to nearly 8% of all currently registered domains (estimated at 359.8 million). Taking subdomain variations into account, the number of distinct account fully qualified domain names (FQDNs) increases to over 29 million, whereas the number of service login FQDNs increases to nearly 8 million unique records, corresponding to 0.75% and 4.36% of the respective domain data. Moreover, with 1076 distinct top-level account domains, the acquired data covers roughly 74% of the currently available 1448 root zone TLDs [28], whereas 1002 top-level service domains cover roughly 69% of the root zone TLDs. Lastly, service login URLs also included over 8 million application deep links using an android:// URL scheme and containing over 161 thousand unique application bundle identifiers amounting to 6.61% of Android applications available on the Google Play Store according to [29]. The most commonly found email account domains in the leaked dataset are given in Figure 6, whereas Figure 7 displays the most commonly found top-level domains, which may, in a limited manner, suggest the geographic distribution of the compromised users. Furthermore, an overview of the most commonly occurring email account domains, service login domains, their TLDs, and application/bundle identifiers is given in Table 4.

### 4.3. Username and Password Reuse

Table 3 reveals that nearly 4 billion records of deduplicated leak data included 1.5 billion (37.9%) distinct usernames, 2.5 billion (61.7%) distinct email addresses, and 1.1 billion (27.5%) distinct passwords, indicating significant password reuse of 72.5%. These formed 2.9 billion (72.6%) unique username and password pairs or nearly 3.8 billion (94.8%) unique email and password combinations. The remaining 200 million records (5.2%) varied in service URLs and application IDs.

**Figure 8:**
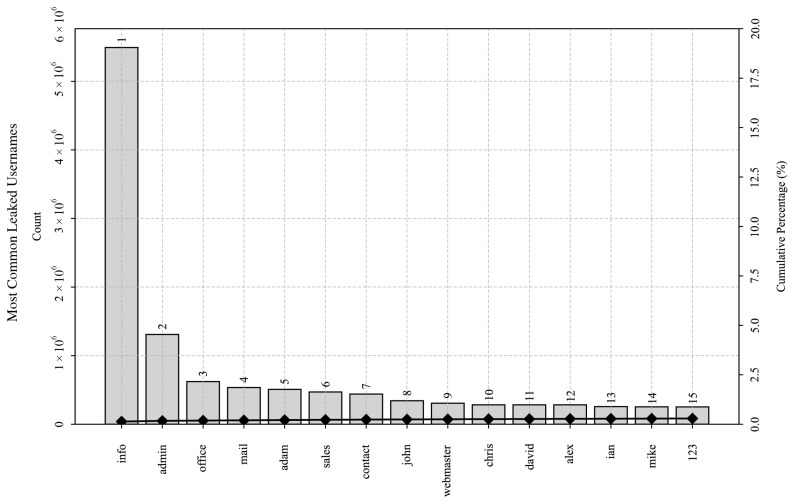
Top 15 most commonly appearing leaked usernames in the 4-billion-row dataset. The chart displays usernames on the vertical axis and their occurrences and cumulative percentages on the horizontal axis. The most common 15 usernames constitute less than 0.5% of all occurrences in the dataset.

**Figure 9:**
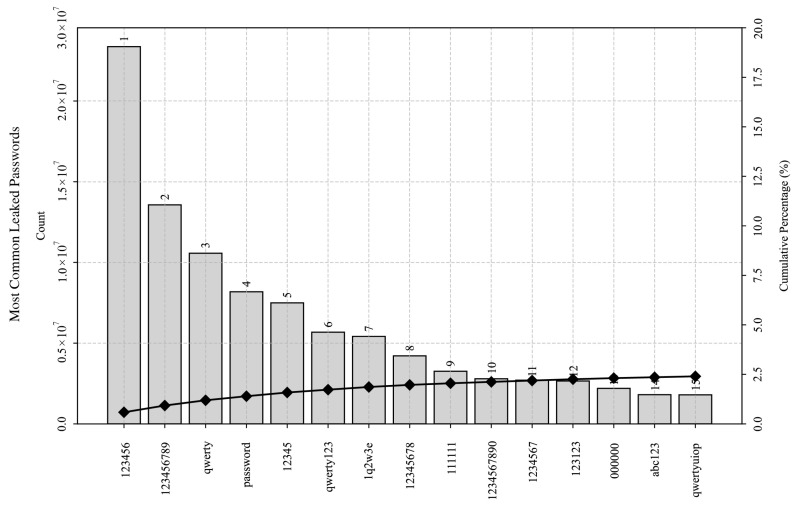
Top 15 most commonly appearing leaked passwords in the 4-billion-row dataset. The chart displays passwords on the vertical axis and their occurrences and cumulative percentages on the horizontal axis. The most common 15 passwords account for 2.5% of all occurrences in the dataset, whereas the top 85,636 passwords constitute 20% of occurrences.

The most common usernames and passwords in the leaked corpora are shown in Figure 8 and Figure 9, respectively. These figures depict a subset of the most commonly occurring usernames and passwords using the Pareto charts, depicting their absolute frequency of occurrence and their cumulative percentage. Despite less than 30% of the passwords in the deduplicated dataset being unique in total, occurrence frequencies of the most common passphrases indicate a power-law distribution with a distinctly long-tail characteristic. Moreover, they also hint at human behavior that tends to favor convenience at the expense of security, as distribution analysis revealed that the top 85,636 passwords constituted 20% of the occurrences in the 4 billion records.

### 4.4. Credential Composition Overview

Syntactic and semantic analysis of common passwords revealed frequent use of repeated sequences (e.g., “123123123”), common character substitutions (e.g., leetspeak, “p4ssw0rd”), and alphanumeric concatenations (e.g., “password123”). These pattern observations directly coalign with National Institute of Standards and Technology (NIST) Digital Identity Guidelines for password security verification [30], and they match the common modes of operation of popular password cracking tools (e.g., mask attack, combinator attack, Markov attack, PRINCE attack, etc.). Furthermore, syntactic and semantic analysis of common passwords highlighted prevalent keywalking patterns, such as “123456,” “qwerty,” “1q2w3e,” and “qwertyuiop.” These patterns are indicative of users’ attempts to create easily memorable passwords by following simple sequences on a keyboard and may also suggest user demographics given their keyboard layout. Similarly, the frequently used (alpha)numeric sequences could potentially hint at a broader cultural or linguistic influence in password creation or reflect practical factors, such as the requirement for switching between input methods in certain Asian languages.

The presence of common usernames such as “info,” “office,” “mail,” “sales,” and “contact” in the leaked dataset indicates that many of the compromised accounts are likely associated with business or organizational functions. Likewise, usernames such as “admin” and “webmaster” likely demonstrate either the presence of leaked default or functional accounts and passwords in the IT infrastructure or breach data contamination, whereas the presence of usernames like “adam,” “john,” “chris,” and “david” suggests that many of the compromised accounts potentially belong to English-speaking users, possibly indicating a demographic concentration of leaked data [31].

We comparatively analyzed leaked passwords and generated keyboard-walking patterns. Our analysis indicates an overlap between the keywalk patterns present in the leaked data and those generated by these tools. Nearly 38% (6126) of the 16,312 generated keywalk patterns matched at least one password in the leaks database, with the cumulative number of matches accounting for nearly 3% of the real passwords under the same criteria (length, TLD). This result is consistent with the observations reported in earlier research [18]. Table 5 lists the most common 15 keywalk patterns occurring in real passwords.

**Figure 10:**
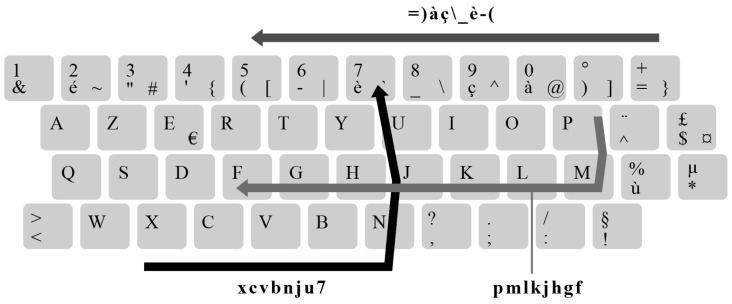
Visualization of three straightforward key-walking patterns matched against known breached passwords on a French AZERTY keyboard layout. The password “=)àç_è-(” consists entirely of special characters and forms a straightforward key-walking pattern without directional changes on an AZERTY keyboard while forming no obvious patterns on alternate keyboard layouts. The password “xcvbnju7” consists of alphanumeric characters and includes one directional change. The password “pmlkjhgf” consists solely of alphanumeric characters and forms a simple keywalking pattern with one direction change on an AZERTY keyboard.

Conversely, we only matched 8 distinct keywalking patterns to 20 leaked passwords when using a Dvorak keyboard layout (excluding numeric patterns and patterns generated on previously analyzed keyboard layouts). Some of the other less commonly occurring patterns include, for example, “=)àç_è-(,” “xcvbnju7,” and “pmlkjhgf,” as visualized on an AZERTY keyboard layout in Figure 10.

This suggests that many users inadvertently create passwords that, while appearing random or complex to them, fall within predictable keywalk patterns and are susceptible to pattern-based cracking methods that leverage tools specifically designed to exploit these tendencies.

We believe these passwords are popular because they are easy for users to remember yet are perceived as secure enough to meet the requirements of various password policies or security metrics. While some password complexity metrics were updated to reflect these weak patterns, others were not. For example, the University of Illinois at Chicago’s Password Meter [32] considers the password “1q2w3e” as “good” and password “1q2w3e4r” as “strong,” despite their apparent patterns and relatively high occurrences in the leaked datasets (5,423,352, and 1,071,387 times, respectively). This highlights a gap between user perceptions of security and actual password robustness.

### 4.5. Prevalent Password Characteristics

**Figure 11:**
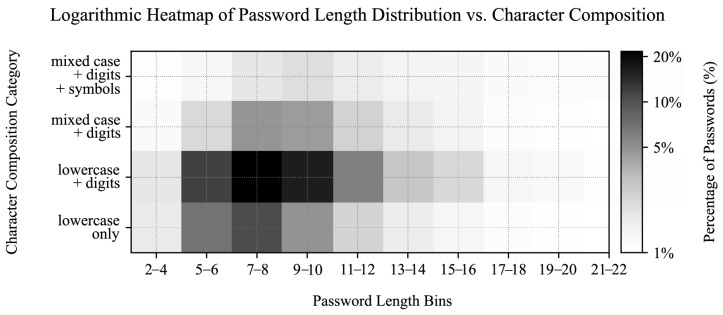
Heatmap showing the distribution of password lengths and character classes in the analyzed leaks dataset. The horizontal axis represents password length bins, whereas the vertical axis categorizes passwords into four classes by character composition. The color intensity, displayed on a logarithmic scale, indicates the percentage of passwords within each category. The heatmap highlights a predominance of shorter passwords with simpler character classes.

In addition to analyzing the most common usernames and passwords, we constructed a heatmap to visualize the distribution of password lengths and character classes. The heatmap in Figure 11 provides a rudimentary insight into how users typically create passwords, focusing on length and character composition. The heatmap categorizes passwords by length (e.g., 5–6, 7–8 characters) and character classes: “lowercase only,” “lowercase + digits,” “mixed case + digits,” and “mixed case + digits + symbols.” The color intensity represents the percentage of passwords in each category, using a logarithmic scale to better highlight variations across the dataset.

Our analysis showed that most passwords are short (most frequently 7 to 8 characters) and often consist of simpler character combinations, such as lowercase letters and digits. More complex combinations are less common, especially as password length increases. This suggests a user preference for simpler, more memorable passwords, which may compromise security. However, while this simple heatmap provides a basic assessment of password complexity, it lacks deeper pattern analysis (e.g., keywalk), checks for common character substitutions and repeated patterns, and does not account for string entropy, indicating a need for more comprehensive security evaluations.

Additionally, we scanned the leaked data corpora for passwords resembling the features and structure of password generation tools. We specifically looked for 20-character passwords, such as gyvqy2-minhyd-rejSeb, consisting of one digit, one uppercase character, and 16 lowercase characters in groups of six consecutive characters, separated by two hyphens, and of 71 bits of entropy, to match passwords generated by the iCloud Keychain’s automatic strong passwords feature [33]. We matched 24,953 leaked passwords belonging to 23,209 distinct usernames and 15,483 distinct emails. The three most common service FQDNs for these accounts included accounts.google.com, www.netflix.com, and www.twitch.tv.

### 4.6. Matching Phone Number Records

Lastly, we analyzed numeric records and phone number occurrences. Approximately 111 million username records consisted solely of numbers and contained nearly 65 million distinct numeric usernames (58.50%). Likewise, almost 600 million numeric-only password records contained nearly 70 million unique numeric passwords (11.29%). Phone number analysis was not as straightforward due to widely differing national numbering plans and (regional) specifics. We pattern-matched a subset of potential distinct usernames and passwords in phone number format and relied on Google’s libphonenumber to attempt to identify valid records. We identified more than 2.2 million usernames and 79 thousand passwords directly conforming to international phone numbers in the E164 format. Additionally, 737,000 usernames and 85,000 passwords exhibited the potential for valid phone numbers when applying minor string substitutions (e.g., replacing the leading “00” with the “+” prefix), whereas 64 million distinct usernames and 55 million distinct passwords exhibited the potential for valid phone number format characteristics when prefixed with correct country codes during iterative attempts. We found nearly 14,000 username phone numbers also appearing in the password column, with over 7000 phone numbers fully representing both username and password in the same row. This amounts to 0.61% reuse of phone number for both username and password (normalized using a total number of unique usernames in form of phone numbers), compared to 2.33% reuse of arbitrary strings for both username and password in the whole deduplicated dataset (normalized using a total number of unique usernames). Out of the latter, more than 6000 records only contained usernames and passwords, whereas the remainder contained an email address in which the username part consisted of the phone numbers in question. In the minority subset of rows where phone numbers represented both the username and password for login, records containing service URLs indicated www.facebook.com, vk.com, and login.live.com as the top three service FQDNs. As reviewed, these services enabled login using phone numbers instead of email addresses.

## 5. HTTP Honeypot Data Analysis

### 5.1. Overview of HTTP Request Data

**Figure 12:**
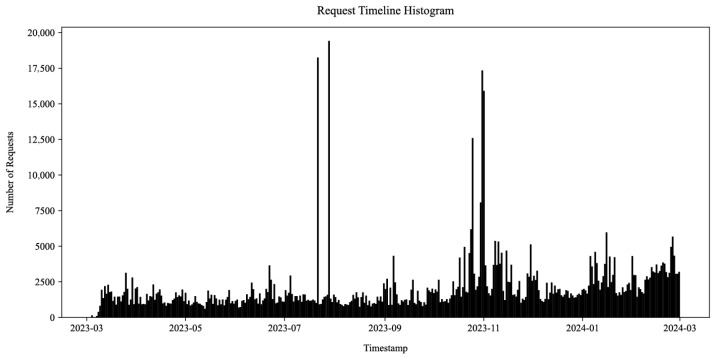
HTTP request timeline histogram. The vertical axis displays the number of received HTTP requests during the time displayed on the horizontal axis. The data was received using 34 HTTP honeypots during 363 days. The histogram displays a baseline honeypot exposure and sporadic high-volume events.

During the course of the experiment, our honeynet captured 754,281 HTTP requests. The requests were received by 34 honeypot sensors across the 12 developed HTTP honeypot services from Table 1. They spanned 363 days and originated from 49,669 unique source IP addresses. Their timeline histogram is shown in Figure 12.

**Figure 13:**
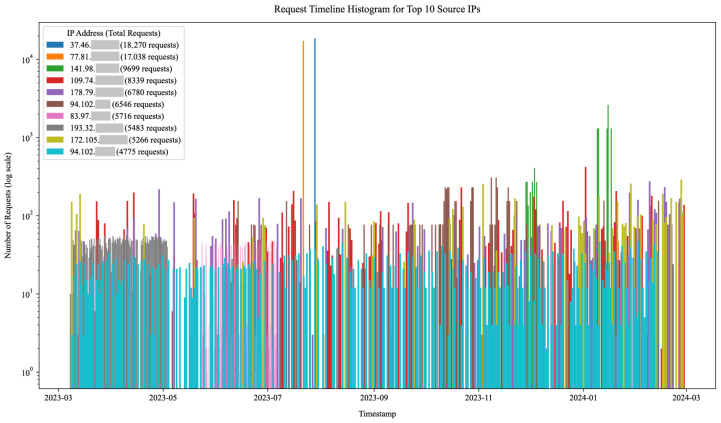
HTTP request timeline histogram for top 10 source IP addresses. The vertical axis represents the request quantity on a logarithmic scale. The horizontal axis displays aggregated request timestamps within 365 bins indicating days. The figure shows that some source IP addresses, such as the one represented in cyan, consistently sent requests to the honeypots, whereas others, like the blue and orange IP addresses, engaged in high-intensity, one-off campaigns. Note the log scale on the vertical axis.

Over 20% of all incoming requests originated from only the 33 most active IP addresses. Our honeypots attributed the incoming requests to 404,398 unique sessions based on the source IP address, timing, referrer, and service model data. The majority of the sessions consisted of only a small number of requests, whereas the top two IP addresses originated over 175,000 requests each. This indicates a long-tail distribution where a small amount of volumetrically significant actors either continuously slowly probed the honeypots throughout the experiment or launched one or more short-lived but high-volume campaigns (e.g., service or vulnerability scanning, login attempts, exploitation, and denial of service attacks). This is visualized in the IP address activity timeline for the top 10 source IP addresses in Figure 13 (log scale). The figure illustrates some source IP addresses continuously sending requests to the honeypots (e.g., year-long activity of the IP address colored cyan) and others launching high-intensity one-off campaigns (e.g., only 15 min traffic bursts from IP addresses colored blue and orange). The latter activity spikes are also visible in Figure 12. Many of these high-profile IP addresses were already flagged as malicious when querying external Cyber Threat Intelligence (CTI) providers.

### 5.2. Attacks Sources and Targets

The IP addresses originated from 3885 autonomous systems and 167 source countries. The most active originating autonomous systems and source countries are displayed in Table 6. Many of the top autonomous system numbers (ASNs) belonged to public cloud service providers, while internet-mapping organizations such as Censys were also observed. The top 10 most active countries by IP geolocation data accounted for nearly 64% of all received requests. Amongst these, the largest number of requests originated from the United States.

Most requests were received by the QNAP NAS honeypots (14.25%), closely followed by phpMyAdmin deployments (13.32%) and Joomla CMS v3 (10.44%). The fewest requests were received by the VMware vCenter Server (2.16%), whereas other honeypot technologies received between 6% and 9% of the traffic.

The vast majority of incoming requests were received on port 80 (98%). Only one percent of the requests initiated secure sessions on port 443, as HTTPS was available only on one honeypot. The remainder of the valid incoming HTTP traffic targeted ports 8080 and 84.

### 5.3. Request and Response Data

The HTTP response codes recorded on the honeypots revealed traffic patterns. The most frequently returned code, 404 Not Found (44.90%), indicated that most of the requests targeted non-existent resources. The second most commonly returned code, 200 OK (42.52%), showed that a significant portion of requests successfully retrieved targets. The remainder of the response codes included 303 See Other, 503 Service Unavailable, 302 Found, 403 Forbidden, 408 Request Timeout, 401 Unauthorized, 405 Method Not Allowed, and 400 Bad Request. These mostly indicated redirection, unauthorized access, client timeouts, and malformed requests. Lastly, unmodeled response code 500 Internal Server Error only appeared once, indicating the stability of the deployed services.

The most common reported client user agent strings included Firefox/62.0 on Ubuntu Linux (8.46%), Chrome/117.0 (8.37%), Chrome/109.0 (5.67%) on Windows, and Safari/602.1 on iPhone14,3 (4.95%). These were followed by curl/7.54 (4.91%) and zgrab/0.x (4.23%). Censys’ CensysInspect/1.1 was the ninth most frequently occurring user agent with 2.69%.

Referrer data in request headers indicated the most referrals from honeypot services themselves (e.g., web resource transfer and subpage requests), although https://www.google.com appeared in 10th place with an occurrence of 1.37%.

Over 82% of incoming HTTP traffic was GET requests, followed by 17% POST requests. The request types OPTIONS, HEAD, PUT, DELETE, and TRACE were also observed but constituted less than 1% of the HTTP traffic altogether.

The five most commonly requested URL paths revealed a significant amount of QNAP common gateway interface authentication attempts (8.13%), scanning for secrets in environment files (2.02%), Optical Network Terminal login and remote code execution attempts likely originating from bandwidth-hungry botnets (1.54%) [34], GeoServer admin panel access attempts (0.64%), and OpenStack authentication attempts (0.59%). Moreover, short bursts of activity visible in Figure 12 in August 2023 revealed repeated database access and export attempts on phpMyAdmin honeypots, but these do not represent a significant portion of the overall traffic.

### 5.4. Attack Source Classification

Based on our actor classification metric [7], we estimate that 19.91% of these requests originated from various internet scanning services we consider as non-malicious. Among the classified IP addresses, 10.39% were flagged as proxies, 0.82% as Tor exit nodes, and 22.61% as VPN users. The distribution of HTTP attackers across honeypots showed that 69.07% of the IP addresses attacked only one honeypot, 12.65% targeted two, and 4.12% attacked three honeypots. The most wide-reaching attacker was a single IP address that accessed 31 honeypots. Lastly, only 297 requests (0.04%) accessed the honeypots using a domain name instead of the IP address. Out of 4 configured honeypot domains, only 2 were referenced, 1 in 289 requests and 1 in 8 requests total.

### 5.5. Authentication Data Analysis

After analyzing the incoming HTTP requests to our honeypot systems, we conducted a thorough review of the HTTP headers, including the URL paths and parameters and the request contents. We parsed the GET parameters found in the URL query strings and any parameters present in the body of the requests (e.g., when POST or PUT methods were used). This allowed us to extract commonly used parameters and their values, which we appropriately decoded where necessary to ensure the correct interpretation of the requests. This analysis aimed to identify potential authentication attempts across different honeypot services.

**Figure 14:**
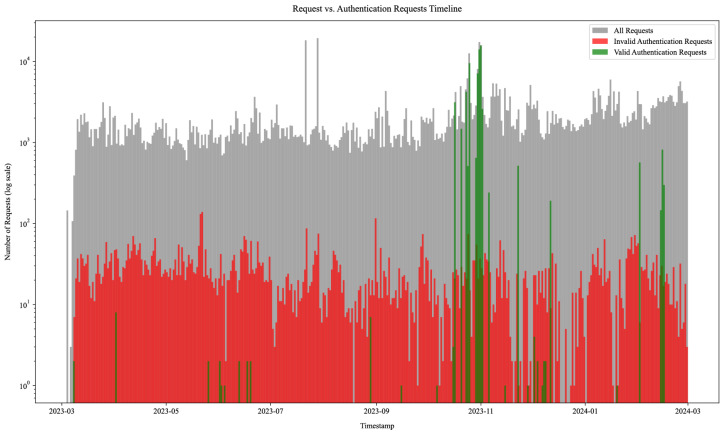
HTTP request timeline histogram depicting request categories. Data in green depict valid authentication requests that conform to the honeypot service models. Data in red depict invalid authentication requests that did not match model requirements (e.g., authentication attempts against an invalid URL, invalid authentication parameters, request method mismatch, etc.). Data in gray depict all incoming requests. Note the log scale.

To facilitate the identification of authentication requests, we built a comparison function that analyzed the URL paths, parameters, and values of the incoming requests against the service models of our honeypots. This function helped determine which HTTP requests represented actual authentication events, such as login attempts or password reset requests. We provide an overview of these authentication requests for all web services, which is illustrated in Figure 14 (note the log scale). The figure categorizes all incoming requests, distinguishing between valid authentication requests (in green) that conformed to the respective honeypot service model, invalid requests indicating login intent but not matching model requirements (in red), and all other requests (in gray). Once again, the figure resembles the request timeline histogram in Figure 12 but offers insight into attack campaigns utilizing tailored requests for performing login attempts on specific targeted services.

Of all 754,281 requests, 127,714 (16.93%) contained a request body, out of which 69,617 (9.23%) contained various login parameters delivered using the HTTP POST method. Additionally, our analysis matched 1089 (0.14%) GET requests that included login parameters in query strings.

In total, we identified 70,706 login requests using both GET and POST parameters, of which 61,819 (87.43%) were deemed valid login requests. Login requests were generated by a relatively small set of 2910 unique IP addresses (5.86% of all unique IP addresses), indicating repeated login attempts from several sources. Out of those, valid login attempts were performed by 1560 unique IP addresses. Table 7 provides an overview of valid and invalid authentication requests by honeypot technology. The invalid authentication requests listed in the table include high-probability matches comprised of either one or more non-service-conformant properties: incorrect URL endpoint paths, incorrect login parameters, invalid parameter value encoding, or invalid HTTP method.

The majority (86.90%) of the invalid authentication attempts appearing on the honeypot services were blind login attempts using /boaform/admin/formLogin URL endpoints. These attempts originated from 1160 unique IP addresses.

### 5.6. Username and Password Analysis

We collected 61,819 usernames (1826 unique) and 61,791 passwords (8386 unique) from all valid login requests and 1070 usernames (67 unique) and 901 passwords (25 unique) from valid login parameters in invalid requests. Additionally, we collected usernames and passwords from semantically named parameters resembling the names of various login parameters in requests that otherwise did not reflect authentication attempts to honeypot services. Altogether, we therefore collected 98,635 usernames and 76,815 passwords, from which 3479 usernames and 8580 passwords were unique.

Table 8 lists the most frequently occurring usernames and passwords detected in all incoming HTTP requests, whereas Table 9 lists the most frequently occurring credentials in valid HTTP login requests.

The honeypot data reveals that attackers frequently attempt access with highly generic usernames like “admin” and “root,” alongside simple, common passwords, including “password,” “12345678,” and well-known wordlist entries. These results are expected, as they align with a brute-force approach targeting default credentials and weak passwords, suggesting that attackers often rely on predictable, easily guessable login attempts to gain unauthorized access. Moreover, when analyzing credential pairs from valid login requests, the username “admin” was most commonly paired with basic passwords such as “admin,” “123456,” “1234,” “password,” “12345,” “123456789,” “1234567,” “12345678,” “000000,” and “111111,” highlighting a clear preference by attackers for default and easily guessable credential combinations. Likewise, we observed a significant number of injection attempts, including command, SQL, and XML External Entity injections, targeting both valid and invalid login parameters.

On the other hand, the least frequently attempted login credentials are perhaps more intriguing than their commonly employed counterparts. They are, however, difficult to discover, as sorting passwords by entropy often results in long keywalking sequences, whereas sorting them solely by length often pollutes the results with command injection attempts. In particular, we recorded only two valid login attempts on the interactive vCenter honeypot, one of which used a unique password that was not found anywhere else online or in common wordlists, not in leaked datasets, and lacked any obvious structural pattern. This prompted us to delve further into analyzing honeypot client-side tracking data and examining login request history per honeypot and per source IP address.

### 5.7. QNAP NAS Login Attack Patterns

**Figure 15:**
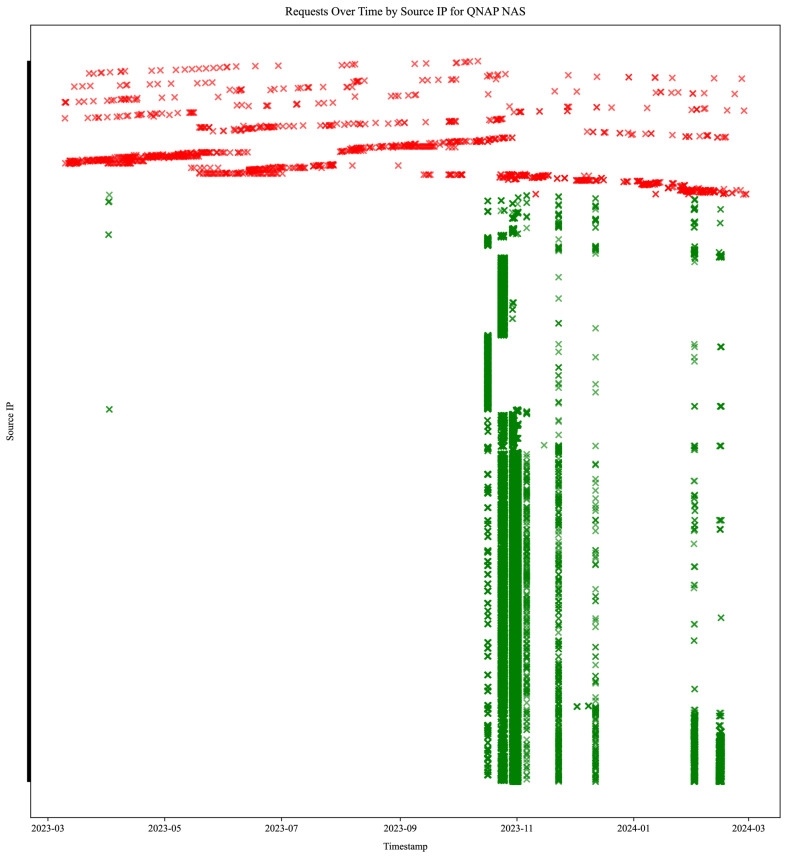
Temporal distribution and origin diversity of 61,574 HTTP login attempts on the QNAP NAS honeypot, distinguishing 1541 valid (green) and 351 invalid (red) source IPs, highlighting distinct attack patterns. None of the IPs originated both valid and invalid login requests to this service, indicating no evolution of attacker specialization.

We visualized login attempts on the QNAP NAS honeypot service, which received the most login requests. Figure 15 illustrates the temporal distribution and origin diversity of login attempts, offering insight into attack patterns against a specific honeypot service. It displays 1892 redacted IP addresses performing 61,574 login attempts. IP addresses performing invalid login request attempts are colored red (351 unique source IP addresses performing 1265 login requests) and span across a wide timeframe. IP addresses performing valid login request attempts are colored green (1541 unique source IP addresses performing 60,309 login requests) in noticeable parallel bursts. None of the IP addresses attacking the QNAP service performed both an invalid and a valid request, whereas seven such addresses were found attacking nine other honeypots (four different services).

## 6. SSH and Telnet Honeypot Data Analysis

### 6.1. Overview of the Collected Events

During the one-year operational period from 1 March 2023 to 1 March 2024, we collected and analyzed extensive SSH and Telnet honeypot data, which comprised a total of 89,219,285 events originating from 422,707 unique IP addresses, with each IP executing 211 events on average. We distinguished between 14 key event types representing various interactions, ranging from password and public key-based login attempts to command executions. Out of all recorded events, 85% of the traffic targeted SSH and 15% targeted the Telnet protocol.

The top 10 ASNs contributing to the traffic accounted for a significant portion of all requests, with Chinanet leading at 28.26%. Other prominent contributors included DigitalOcean (8.28%), Tencent Building (4.42%), and Shenzhen Tencent Computer Systems Company Limited (3.86%). These ASNs, along with the most common countries of origin, are shown in Table 10. Out of the detected source IP addresses, 1.75% (7385 IPs) were marked as known proxies, 0.05% (196 IPs) as Tor exit nodes, and 1.30% (5478 IPs) as VPN servers. We also marked 18,249 (4.32%) originating IP addresses as abusers, given their past malicious activity on the honeynet. However, it should be noted again that many IP addresses may have also served as proxy hosts or VPN servers, thus obfuscating the true origins of the attacks and making it challenging to accurately attribute the activity to specific regions, countries, or organizations.

### 6.2. Analysis of Executed Commands

The dataset revealed over 18 million login attempts, about 5% of which were successful. Each successful login enabled the attackers to execute commands in proxied Linux environments. We captured command inputs and keystroke timing data during the (interactive) sessions for behavioral analysis of attackers and for acquiring insights into automated exploitation tools versus manual human interactions. Roughly 44.20% (361,644) of all successful logins were followed by at least one shell command. The distribution of the number of entered commands per session showed a heavy bias towards a small number of entries, with the vast majority of the sessions only executing between five and ten commands with extremely short delays, indicating repetitive attacks using automated exploitation scripts. On average, each session consisted of eight command inputs and lasted for 29.22 s, with the commands entered 0.07 s apart, thus indicating the prevalence of automated scripts. The longest sessions were over 18 min long, and the longest delay between two consecutive command inputs was nearly 16 min. The total number of commands executed was 3,000,700 (3.36% of all captured events).

The attacker with the most executed commands attempted executing 105,251 commands within 10,526 sessions, amounting to 3.51% of all executed commands. The top 10 most active attacker IP addresses executed 405,060 commands within 41,051 sessions, amounting to 13.50% of all executed commands. The most commonly attempted commands, sh, shell, enable, and system, indicated attempts at obtaining shell environments and elevating privileges on various devices. The enable command either enabled shell built-ins in scripts targeting Linux environments or likely targeted privileged exec mode on Cisco IOS devices in others, whereas the system command is often associated with attempting to execute actions on MikroTik devices. Some non-standard commands were not implemented on our Linux-based honeypots, and their invocation failed. The remainder of the commonly attempted commands included environment reconnaissance attempts, connectivity checks, and file operations (typically attempting to download remote payloads and maintain persistent access). Among the listed examples, we also detected numerous miner installation attempts, Dota3 malware indicators of compromise, mysterious widespread campaigns, such as the “oinasf” script, and frequent access to the authorized_keys file. Besides common scripts, numerous commands were chained with logical operators to address multiple environments and aid their chances of successful execution. These statistics generally reveal a lack of attacker specialization and their wide reliance on generic, automated scripts designed to target a broad range of devices and systems without tailoring for specific environments.

### 6.3. Authentication Attempts and Sources

In total, the dataset revealed 17,866,502 username-and-password-based login attempts, of which 17,048,385 (95.42%) failed and 818,117 (4.58%) were successful. Login attempts were made from 174,816 unique IP addresses, originating from 9855 ASNs in 215 countries. Successful logins were highly concentrated among a subset of IP addresses, with 72,110 IPs (17.06% of unique IPs) responsible for all successful attempts. On average, each IP address made over 165 login attempts. The most active IP address made 737,847 login attempts, whereas the top 10 attackers combined made 3,301,471 attempts (4.08% and 18.24% of total attempts, respectively).

### 6.4. Usernames, Passwords, and Keys

Nearly 18 million username-and-password-based SSH/Telnet login attempts contained only 99,398 unique usernames (0.55% of all received usernames) and 260,911 unique passwords (1.46% of all received passwords), indicating significant credentials reuse during authentication attempts. On average, 4 different passwords were attempted per username, and 19 different usernames and 68 different passwords were attempted per source IP. The most active IP addresses each attempted 2726 different usernames and 33,716 different passwords within 10,670 and 59,970 sessions, respectively. The most common usernames, passwords, and their combinations are listed in Table 11.

Additionally, we recorded 311,987 TCP/IP tunneling requests over SSH, as well as 52,826 SSH key-based authentication requests. The latter only used 157 unique public keys (0.30% of all), originated from 2582 unique IP addresses, and attempted login with 235 unique usernames. On average, each key-based authentication request attempted three different usernames per key, and each IP attempted five different keys. The most active IP address attempted key-based login more than 7116 times with 52 different keys. The most commonly attempted usernames during key-based authentication are listed in Table 12.

### 6.5. Client Fingerprinting and Clustering

We identified 144,741 unique client versions, with “SSH-2.0-Go” appearing the most commonly (6,037,547 occurrences). A list of the top 10 most commonly utilized SSH/Telnet clients is shown in Table 13. The data reflect the dominance of automated tools, scripts, and programming libraries in attack scenarios. On average, each client version was used nearly 95 times, whereas each IP address used an average of 3 unique client versions. The IP address with the largest number of different client versions reported 77,306 unique client strings by appending IP address and number patterns to the “FlowSshNet_SftpStress” string. These results exhibit limited potential for preemptive SSH filtering based on client fingerprints and version strings—particularly those associated with automation tools or non-standard clients—offering an additional defense layer beyond IP-based blocking.

Some of the reported version strings were non-conformant to RFC 4253 specifications (17,762 instances). Similar to the findings in [35,36], these included various HTTP request strings, Réseaux IP Européens (RIPE) Atlas and Stretchoid related probes beginning with MGLNDD_* pattern, XMRig and Open Ethereum Mining Pool JSON RPC calls, as well as shellcode injection attempts.

We used sentence transformers to generate embeddings and k-means to cluster the remaining non-conformant and pre-SSH-2.0 version strings into six categories: HTTP requests (129 unique strings), Shellcode injections (85 unique), Proxy protocol requests (70 unique), RIPE Atlas and Stretchoid probes (31 requests), shell commands and other protocols (29 unique), and Pre-SSH-2.0 clients (25 unique), including some Nmap Scripting Engine strings and NXSH records likely belonging to Shodan.

Lastly, we compared the captured HASSHes against a database of 2017 known SSH tools in various configurations and matched 144 entries. The most common client versions largely remained the same and included the Go SSH library, libssh, AsyncSSH, and PuTTY. Moreover, we detected numerous instances of ZGrab and Nmap scans, as well as the common use of Rust, Java, and JavaScript SSH libraries. We also detected frequent activity of RapperBot, an SSH-brute-forcing IoT malware adapted from Mirai source code and utilizing hardcoded common username and password values, as well as client configuration commonly associated with Hydra and Metasploit.

## 7. Approaches Towards Data Cross-Examination

### 7.1. Cross-Service Attack Overlap

Throughout this study, we analyzed billions of leaked credentials and extensive attack data collected from honeypots in an isolated manner. The results revealed widespread credential reuse, persistent login attempts with weak credentials, and automation-driven attacks from a small pool of active IP addresses. Notably, 19.79% (9828 IP addresses) of attackers who targeted HTTP services also attempted to access SSH (2.12% of all unique source IP addresses detected on the honeynet). Among these, we classified 39.48% (3880 IPs) as known scanners, 23.93% (2352 IPs) as proxies, 0.80% (79 IPs) as Tor exit nodes, and 35.24% (3463 IPs) as VPN users. We also marked 5455 (55.50%) of these IP addresses as known abusers due to their previous actions detected on our honeynet and network telescope. Together, these attackers accessed 37 HTTP deployments and 31 SSH/Telnet honeypots in all 3 deployment sectors (cloud, academic, and critical infrastructure).

### 7.2. Credential Composition Analysis

We previously categorized leaked passwords into character composition groups and sorted them by length across their sources in Section 4.5. We repeated the analysis for other datasets to enable their cross-comparison.

**Figure 16:**
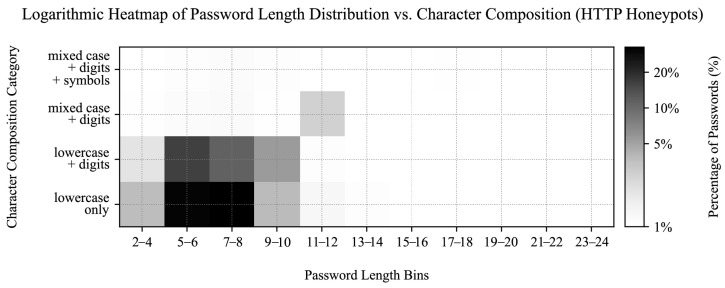
Heatmap visualizing the distribution of password lengths and character classes of passwords captured during the valid authentication requests against HTTP honeypot services. The horizontal axis represents password length bins, whereas the vertical axis categorizes passwords into four classes by character composition. The color intensity, displayed on a logarithmic scale, indicates the percentage of passwords within each category.

Amongst the valid HTTP login attempts, most of the passwords were 7–8 characters long and consisted of lowercase letters (31.53%), followed by 5–6 character passwords of lowercase letters (29.54%) and lowercase letters with digits (15.24%), as shown in Figure 16. Only 10.07% of valid HTTP login attempts matched the most common group from the real-world leaks dataset (7–8 character lowercase letters and digits), whereas 9–10 character passwords of similar composition accounted for just 4.38%. When reviewing invalid HTTP login attempts (e.g., unexpectedly base64-encoded passwords or injection attempts), we found longer and more complex passwords to be more frequent. On the other hand, shorter and simpler passwords were prevalent among the credentials provided via the HTTP GET method in supporting services.

**Figure 17:**
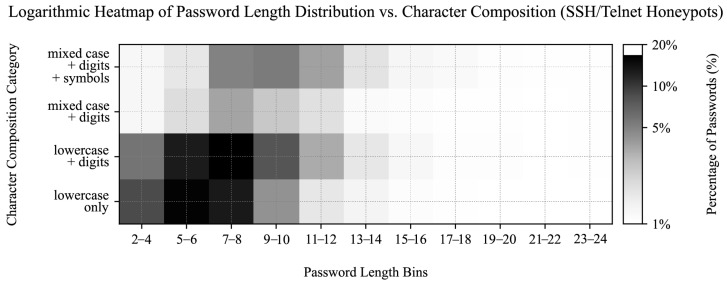
Heatmap showing the distribution of password lengths and character classes of passwords captured during SSH and Telnet honeypot authentication attempts. The horizontal axis represents password length bins, whereas the vertical axis categorizes passwords into four classes by character composition. The color intensity, displayed on a logarithmic scale, indicates the percentage of passwords within each category.

SSH and Telnet credentials aligned more closely with the leaked dataset in terms of password length and character composition, with 15.12% of attempted passwords consisting of 7–8 character lowercase letters and digits, followed by 5–6 character lowercase-only passwords (14.89%), 7–8 character lowercase-only passwords (11.97%), and 5–6 character lowercase letters with digits (11.63%), as shown in Figure 17. Additionally, SSH and Telnet passwords showed greater dispersion across the characteristic groups compared to HTTP attempts.

While the above results suggest an apparent lower adaptability of HTTP attackers to real-world password datasets, it is important to highlight the potential bias, as well as the diversity of leaked data due to its numerous sources and its possible contamination (e.g., with the default username and password combinations from various vendors or service providers). For this reason, the attackers may have already specialized their attacks using subsets of likely credentials for particular services. These subsets of credentials may feature characteristics different from the generalized review of password length and composition in the whole leaked dataset or combined passwords across all HTTP services. This called for a further per-service examination of attack credentials.

### 7.3. Significance of Password Variety

We first assessed the statistical significance of password composition variety between the SSH/Telnet, HTTP (valid requests only), and leaked password datasets (Table 14). We performed pairwise chi-square tests on normalized percentual category data between the samples, as visualized in Figure 11, Figure 16 and Figure 17.

The null hypothesis for each comparison was that the distributions of password lengths and character composition categories were the same between the datasets. In this case, the variables under comparison were password length bins and character composition categories, totaling 54 degrees of freedom (df).

In all comparisons, the *p*-value (effectively 0.0) was well below the significance level α, indicating that the observed differences between the datasets were statistically significant. However, due to the test’s sensitivity to large sample sizes, we also decided to estimate the effect sizes using Cramér’s V ($C_{V}$), which were nearly negligible.

Next, we reviewed the password composition variety between the honeypot deployment sectors (critical infrastructure, academic institutions, and cloud services) for HTTP and SSH/Telnet honeypots (Table 15).

The most substantial differences were found when comparing the HTTP dataset against the others. For the critical vs. academic comparison (HTTP: $\chi^{2}=11.57,\;p=0.9897,\;df=25$, SSH/Telnet: $\chi^{2}=2.54,\;p=1.0,\;df=43$) and critical vs. cloud comparison (HTTP: $\chi^{2}=14.19,\;p=0.9705,\;df=26$, SSH/Telnet: $\chi^{2}=1.73,\;p=1.0,\;df=43$), the high p-values indicated a failure to reject the null hypothesis, supported by negligible effect sizes with Cramér’s V values. Similarly, the academic vs. cloud deployment comparison (HTTP: $\chi^{2}=0.77,\;p=1.0000,\;df=26$, SSH/Telnet: $\chi^{2}=7.51,\;p=1.0,\;df=43$) showed almost identical distributions, with a Cramér’s V of 0.0172.

However, the datasets varied significantly in size, and large sample sizes can impact the test’s sensitivity to small variations. While we normalized the data to mitigate some of these concerns, the high degrees of freedom and uniformly high *p*-values indicate that such tests may have limited utility in distinguishing subtle differences between password distributions. Moreover, the assumption of independence among observations may not hold entirely, given the large potential for password reuse across attacks.

In terms of size, HTTP honeypots in the critical sector collected 632 passwords, compared to 30,152 and 31,007 passwords collected by HTTP honeypots in academic and cloud deployments, respectively. SSH and Telnet honeypots in academic environments collected 11,518,543 passwords, whereas SSH and Telnet honeypot cloud and critical sector deployments collected 5,293,968 and 1,057,708 passwords, respectively.

We conclude that while the datasets differ in their password length and composition patterns in inter-protocol deployments (e.g., SSH vs. HTTP) and in comparison with leaked data, the statistical analysis revealed no significant variations in password composition between any pair of sectors of the intra-protocol honeypot deployments (e.g., SSH/Telnet critical vs. SSH/Telnet academic, or HTTP critical vs. HTTP cloud, etc.). This, in turn, reflects the potential variations in the behavior and preferences of attackers (e.g., employment of different credentials stuffing wordlists and brute-force methods) when targeting different services but indicates similar password-guessing strategies across the honeypots of the same type (e.g., focusing on weak or systematic passwords), regardless of the targeted sector.

### 7.4. Dictionary, Leak, and Wordlist Coverage

To address the limitations of the above statistical tests, we focused on examining the origins of the usernames and passwords used in these attacks. We compared the collected datasets and introduced well-known wordlists and default account credentials to understand the the prevalence of dictionary entries and default (device or service) credentials in the real world, as well as the influence of widely circulated breached data on attacker strategies. Moreover, such comparisons may also allow us to attempt to indirectly estimate breached data contamination.

We compared the leaked data and honeypot password datasets against the preprocessed and deduplicated OPTED, WordNet, and Wiktionary (English) dictionaries, containing 111,597, 147,478, and 1,269,204 unique entries, respectively. We provided the diversity (deduplicated) and frequency (non-deduplicated) cross-comparisons between the dictionaries and the credential datasets in terms of full string matches as percentual coverages of datasets and Jaccard indices, also known as Intersection over Union (IoU). The results are displayed in Table 16 (usernames) and in Table 17 (passwords).

The results highlight the overlap between some of the collected credential datasets and modern dictionary-based wordlists, emphasizing the role of common words in username and password choices. For HTTP usernames, 37.95% of all unique username entries attempted during honeypot logins also appeared in the English Wiktionary. However, these entries only represented 0.05% of all unique words existing in the English Wiktionary. Moreover, the data reveal that many username attempts were often repeated, with 63.60% of total (non-unique) attempted username entries also matching an English Wiktionary entry.

SSH/Telnet usernames also exhibited great reuse, with 86.85% of all attempted usernames on honeypots matching an English Wiktionary entry. However, when deduplicated, only around 10.86% of unique SSH/Telnet usernames matched a word in the Wiktionary, covering 0.85% of its total entries (or over 5.02% of all WordNet entries). In both cases, the coverages are much smaller when the username sets are compared to the smaller WordNet dictionary and practically non-existent when compared to the much older OPTED dataset, indicating substantial differences between the dictionaries and their relevance.

Inversely, a large dataset of usernames from breached databases only matched 0.03% of its unique usernames against the Wiktionary, yet these matches covered nearly 37.50% of all words in the dictionary. While this inversion is expected due to the very large differences in dataset sizes, it also visualizes the frequency of dictionary-based attacks while at the same time showcasing their practical limits (in terms of small joint dictionary coverage during the full one-year of the attacks) or reveals the attacker wordlist specialization (e.g., many attackers employing only a select subset of dictionary words).

The calculated IoU values are given as a provisional metric for the similarity of unique sets. Due to the vast differences in dataset sizes, these values are very low, limiting the metric’s usefulness and indicating a large diversity between sets.

The password datasets followed a similar pattern, with 26.69% of unique HTTP passwords appearing in Wiktionary and covering 0.18% of its contents. SSH/Telnet passwords showed lower direct overlap but still exhibited 20.54% Wiktionary and 16.5% WordNet matches of the total password attempts. The leaked passwords had a substantial presence in the dictionaries, with 50.19% coverage of WordNet and 32.12% coverage of Wiktionary. The inverted coverages again indicate that the attackers frequently rely on partial subsets of English dictionaries to perform their attacks (or are unable to test more of dictionaries’ contents), whereas users, on the other hand, seldom employ unmodified dictionary words as passwords (yet cover much larger portions of dictionaries when doing so). We provided the passwords from all three datasets that most frequently matched the Wiktionary entries (Table 18). Matches against some passwords, such as “qwerty,” “qwertyuiop,” and “asdfghjkl,” indicate the wide span of word entries in the Wiktionary.

We also compared the collected datasets to the Russian and German Wiktionaries. We extracted 466,924 unique words in Cyrillic from a Russian Wiktionary and 871,444 unique words from a German one. Set coverages and IoU metrics for these comparisons are given in Table 19 and Table 20. Non-deduplicated dataset comparisons were skipped where set intersections contained no elements. Also note that the English, Russian, and German Wiktionaries contained some intersecting elements (English ∩ Russian: 2 elements, IoU: 0.00, coverages: 0%; English ∩ German: 15,182 elements; IoU: 0.01, coverages < 1.75%; German ∩ Russian: 1 element, IoU: 0.00, coverages: 0%).

Next, we compared the collected credentials against five widely used wordlists: FastTrack (262 entries), a small set from the Social-Engineer Toolkit; John The Ripper (3545), focused on weak and common passwords; Nmap (4999), containing default credentials for network services; RockYou (14,344,392), a massive dataset from a well-known breach; and Wifite (203,808), targeting likely Wi-Fi and general authentication passwords.

Instead of opting for heavier collections (e.g., SecLists, Weakpass, and Probable Wordlists), each of these wordlists provided a unique perspective, ranging from small and targeted lists optimized for penetration testing (FastTrack, John, Nmap) to larger datasets derived from real-world password leaks (RockYou). Cross-comparison results are given in Table 21. They indicate that widely used wordlists, such as John, Nmap, and RockYou, capture a significant portion of real-world passwords, with up to 100% of their entries appearing in leaked datasets. HTTP passwords show particularly high overlap with John (25.4% coverage, 0.22 IoU) and Nmap (58.06% coverage, 0.57 IoU), suggesting attackers frequently rely on these lists. SSH passwords have lower overall coverage, reflecting more variation in credential guessing.

We also compared the datasets with the default credential lists (pairs of usernames and passwords) obtained from sources listed in Section 3.3. The comparison results are given in Table 22.

These results reveal that credential pairs from the selected default account lists were used when attacking SSH, Telnet, and HTTP honeypots. Notably, the Mirai account list was used in its entirety when attempting connections to the SSH/Telnet honeypots. However, the A coverage values suggest that these lists were too limited, as attackers tested many other credential combinations as well. Similarly, while various well-known default credentials appeared in the leaked dataset, they accounted only for a small and insignificant fraction of the overall data.

Moreover, a detailed examination of the Nessus vulnerability scanner’s account check plugin revealed that the tool attempts to perform SSH and Telnet logins using randomly generated credentials of eight characters in length to assess whether the remote system accepts arbitrary login attempts. The plugin’s description attributes this check to the prevention of false positives, explicitly referencing Mocana SSH within the code. However, we hypothesize that this functionality may also serve as a rudimentary mechanism for identifying less sophisticated honeypots.

Lastly, we directly compared the HTTP, SSH, and Leaks datasets against each other. We compared full-string matches between usernames, passwords, and credential pairs. When comparing username data with leaks that included email addresses, we extracted the username handles from the emails and compared those directly.

The results in Table 23 reveal that 90.35% of usernames and 72.52% of passwords used in HTTP attacks have been reused in attacks on SSH or Telnet honeypots. Moreover, 95.97% of usernames used in HTTP attacks and 83.45% of usernames found in SSH/Telnet attacks, as well as 91.27% of passwords found in HTTP attacks and 67.40% passwords found in SSH/Telnet attacks, also existed as entries in the leaked credentials database. However, these numbers were significantly lower when comparing credential pairs, where only 16.18% of HTTP username and password pairs were also used in SSH/Telnet attacks, 65.91% of HTTP accounts, and 25.24% of SSH/Telnet accounts also existed in leaked databases. The total number of attempted unique HTTP and SSH/Telnet credential pairs amounted to less than 0.01% of all unique accounts in the leaked database.

### 7.5. Personal Information, Patterns, and Mutations

We used a regular-expression-based approach to identify the potential personally identifiable information in the honeypot-collected datasets. Focusing primarily on email addresses and phone numbers, we identified 86 credential pairs in the SSH/Telnet dataset where the username followed a valid email address format. In contrast, no email-like usernames were found in the HTTP dataset.

Among the matched SSH/Telnet account email addresses, 34 were unique, and each contained a distinct username handle. In total, the addresses were hosted on five different domains, all of which were registered under the .com TLD. The domain with the most email address matches appeared with 30 unique username handles, whereas the remaining 4 domains only had a single occurrence each. Only one of the addresses belonged to a widely recognized email provider, gmail.com.

To refine our domain-based matching of these accounts against the leaked credentials database, we began by excluding the gmail.com match (as we already knew that numerous Gmail accounts were present in the leaks) and analyzed the remaining four domains. This process revealed 2704 email address entries across 2 of the 4 remaining domains.

Importantly, we found one email address that was used as a login username in SSH honeypot attacks and was also present in the leaked credential database. This particular address appeared with five different password entries in the leaks database, none of which matched the three passwords attempted during the SSH logins. Notably, the attempted SSH passwords followed a string concatenation pattern, where numerical digits were appended to a base loweralpha password.

Furthermore, to match the potential phone numbers appearing in the submitted HTTP and SSH/Telnet passwords, we applied the same detection methodology as outlined in Section 4.6. Out of all received unique usernames, 1.25% of HTTP and 0.19% of SSH/Telnet usernames consisted solely of digits. For passwords, this amounted to 4.25% and 6.02% for HTTP and SSH/Telnet, respectively. These percentages stayed consistent when including other potential number formatting characters, such as space, dash, and plus symbol in the regex pattern. Using Google’s libphonenumber, we did not find any phone numbers directly conforming to the E164 format. However, we did match 11 usernames and 301 passwords amongst the HTTP dataset and 948 usernames and 23,395 passwords amongst the SSH/Telnet dataset, which exhibited the potential for valid phone numbers when prefixed with the correct country codes and/or applying minor formatting substitutions.

When comparing the keywalk patterns generated in Section 4.4 with the HTTP and SSH/Telnet credentials datasets, we discovered that 0.87% of unique HTTP usernames and 0.83% of unique HTTP passwords matched a keywalk pattern. For the SSH/Telnet datasets, these numbers amounted to 0.5% of distinct usernames and 0.28% of distinct passwords. For Dvorak keyboard layouts, we matched 0.60% of HTTP usernames, 0.44% of HTTP passwords, 0.29% of SSH/Telnet usernames, and 0.13% of SSH/Telnet passwords. Inversely, only 0.44% of all generated keywalk patterns were found in the valid HTTP passwords dataset and only 4.53% in the SSH/Telnet password dataset. This indicates a significant drop in keywalk coverage compared to the 38% of all generated keywalks found amongst the leaked passwords. In terms of attempt frequency, the most commonly attempted HTTP password keywalk patterns are listed in Table 24, and the SSH/Telnet passwords followed a rather similar distribution. For usernames, the majority of keywalking matches were only two (HTTP) to three (SSH/Telnet) characters in length, making them unlikely username choices. For passwords, the average matched keywalk pattern length was seven characters, closely resembling the length of real-world passwords.

**Figure 18:**
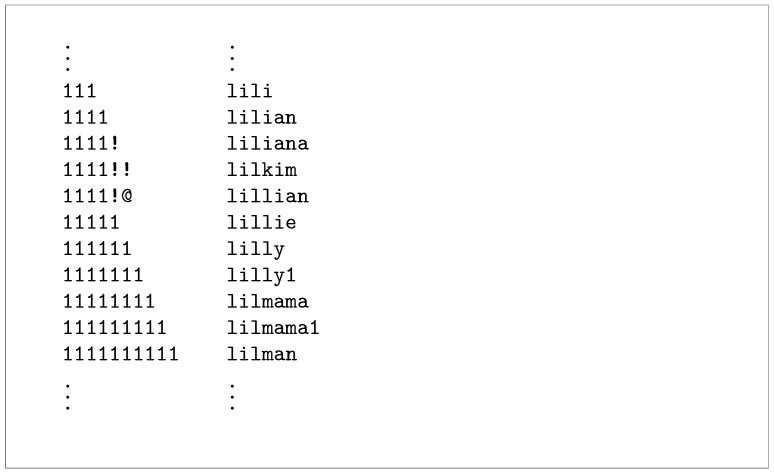
Trivial example of brute-force and wordlist-based password attacks showing iterative password retries using alphabetic sorting of passwords.

When reviewing password patterns, simple alphabetic sorting of all joint attempts captured on honeypots trivially reveals the generalized brute-force nature of the attacks (sorting excerpt in Figure 18). However, such an approach hides the needle in the haystack even further, as it generalizes the attack data instead of focusing on specific attack characteristics, as in the previous analyses.

Additionally, we attempted to perform a rudimentary search-based password analysis to reveal any potential correlation between the user’s and attacker’s password choices and major world events. First, we reviewed how many usernames and passwords contained numbers representing years from 1950 to 2025 as substrings. In the HTTP dataset, 126 (0.20%) attempted login passwords (47 unique) contained a total of 37 unique year substrings. The majority of these matches consisted solely of digits, and only a few contained years appended to strings. The most commonly appearing was the year 2000, with 25 matches. The latest year in the dataset was 2020, with 13 occurrences. We did not find any year substrings in the HTTP usernames dataset, but we did find 5680 (0.03%) usernames containing year strings in the SSH/Telnet dataset (572 unique username values, 46 unique year numbers). The most commonly appearing year in the latter was 2015 (2674 appearances), followed by 2000 (786 appearances) and 2018 (369 appearances). The latest appearing year was 2024, with 15 occurrences. The vast majority of appearances were in the form of the concatenation of the year numbers to a loweralpha string.

Much larger year substring numbers were observed when analyzing SSH/Telnet passwords, where 5.94% of all passwords (1,075,788 attempts, 16,621 unique passwords) included a year number (spanning across all 76 years). Here, the most common substrings were 1987, 1986, and 1988, each with roughly 78,000 occurrences. The latest year was again 2025, with 419 occurrences. However, this large subset of matches only contained 126 entries (47 unique passwords) where the characters adjacent to year numbers were not digits, thus indicating that the majority of these matches came from purely numerical passwords. Moreover, only 95 attempts (37 unique passwords) contained four-digit-long years appended to the end of the string and 90 attempts (33 unique passwords) at the beginning. Out of the former, 16 unique passwords contained only the loweralpha character set with the year number appended, whereas out of the latter, only 12 prepended the year to a loweralpha-only string.

In the leaks database, we matched all 76-year substrings in 127,171,129 unique usernames (8.39%) and 88,446,354 unique passwords (8.02%). The vast majority of these credentials again included year matches with non-digit adjacent characters. From the leaked passwords dataset, 202,932,518 (5.07%) unique records were comprised of loweralpha characters and an appended year.

Next, we compared the leaks database against the top 230 Google Trends from the last decade (worldwide trending search keywords, case-insensitive, five characters or longer). There were very few substring matches amongst the attempted (non-unique) HTTP passwords. The top 10 included the following: “apple” (49 matches), “pokemon” (14), “hotmail” (14), “yahoo” (13), “samsung” (10), “google” (10), “lakers” (9), “cricket” (8), “youtube” (4), and “kickass” (4). Amongst the SSH/Telnet passwords, the most common substring match was again “apple” (14,834 attempts), followed by “google” (14,537), “video” (11,522), “samsung” (4589), “yahoo” (2227), “pokemon” (2159), “facebook” (1340), “lakers” (1246), “weather” (1060), and “amazon” (1034). There were also 322 username attempts containing “trump,” 163 containing “putin,” 63 containing “ukraine,” 60 containing “tiktok,” and 1 containing “coronavirus.” Amongst the HTTP usernames, the dataset only contained three login attempts with substring matches for “apple,” two attempts matching “samsung,” “video,” and “bing,” and one attempt containing “mail.” Amongst the SSH/Telnet usernames, the most common substring match was “games” (1838 occurrences), followed by “apple” (1470), “video” (147), “google” (113), “pokemon” (99), “tiktok” (70), “amazon” (52), “satta” (46), “samsung” (45), and “weather” (45). We also detected 12 login attempts with usernames containing “trump,” 5 containing “putin,” 2 containing “ukraine” and 1 containing “shein.” In the leaked username dataset, the most common substring match was “apple” (1,175,393 occurrences), followed by “games” (1,150,179), “google” (731,909), “yandex” (571,491), “video” (490,154), “facebook” (449,815), “amazon” (354,012), “yahoo” (347,768), “gmail” (317,825), and “hotmail” (302,382). We also detected relatively high occurrences of “pokemon” (212,049), “roblox” (200,554), “putin” (183,290), and “twitter” (152,849), whereas “craigslist” (11,744) “coronavirus” (712), and “chatgpt” (102) were matched far less often. In the leaked passwords dataset, the most frequently occurring substring was “gmail” (17,340,500 occurrences), followed by “hotmail” (16,149,820), “yahoo” (15,801,444), “apple” (1,967,599), “google” (1,235,855), “samsung” (1,204,717), “yandex” (1,200,807), “pokemon” (975,101), “facebook” (691,428), and “outlook” (640,507). “Putin” was a substring in passwords 83,876 times, “fortnite,” “ukraine,” and “deadpool” rounded off around 53,000 times, whereas “coronavirus” and “covid-19” were mentioned less than 3000 times. Given the obvious presence of product and service names and common abbreviations of Google searches in account passwords, we also compared e-mail address domains with passwords, where 15,221,988 unique records included the word “hotmail” both in the e-mail address domain as well as in the user’s password. This number was much lower for the substrings “facebook” (41,060), “apple” (7819), and “youtube” (810). The data suggested that the trends observed in the leaked data could potentially be leveraged to approximate the age and relevance of compromised data, albeit in a limited manner.

Further, we reviewed the incidence of password mutation (e.g., leetspeak, concatenation, etc.) by comparing the most common dictionary passwords against the rest of the dataset entries and calculating their edit distance. We compared select Wiktionary matches from the top 10 leaked passwords that were not considered keywalk patterns. We based the metric on Levenshtein distance to allow for variability in string length and to handle substitutions, as well as insertions, deletions, and transpositions, and we normalized it by the maximum length of either password to approximate the proportional similarity score. We then defined a 35% change as a cut-off point for basic mutations (as larger values often began to show base word shifts) and compared the results between HTTP, SSH/Telnet, and leaks datasets. Table 25 reveals the incidence of password mutation for the words “password,” “monkey,” and “killer,” alongside the samples of the most common mutated passwords inside their metric bins.

While password recovery software, such as hashcat, already features predefined mutation rulesets (e.g., best64, rockyou-30000, d3ad0ne, etc.), we believe that our results, when extended, could help generate new rules that are better optimized for real-world passwords (using newer and larger datasets in comparison to rockyou). Moreover, we believe that it is also viable to use our password datasets with neural network-based password generation approaches that could yield improvements in optimized password guessing, particularly given that previous studies have reported a scarcity of training data [22].

**Figure 19:**
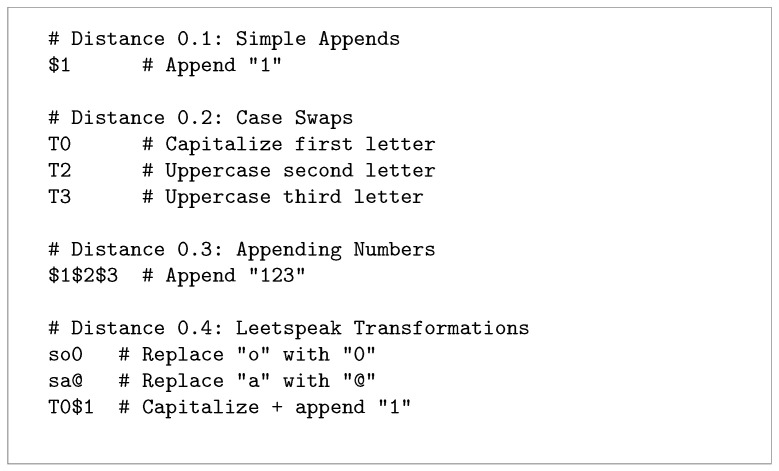
Elementary hashcat password mutation ruleset. The comments must be removed so hashcat can parse the rules correctly.

For demonstrational purposes, we are attaching a rudimentary hashcat ruleset for producing mutated passwords based on the occurrence frequency of the top password mutations within the real-world leaks dataset, as shown in Table 25. The rules in Figure 19 are derived from the top mutations from the first three bins (allowing up to 35% modification) of the word “password”.

## 8. Conclusions and Future Work

Authentication is the cornerstone of security in modern information and communication systems. While asymmetric mechanisms such as public key authentication, as well as WebAuthn, passkeys, and multi-factor authentication, offer robust security solutions, many services continue to allow password-only authentication. Consequently, millions of users rely solely on usernames and passwords, exposing themselves to credential-based attacks and increasing their security risks.

This also increases the chances of success for attackers relying on phishing and social engineering. Furthermore, it exacerbates the impact when digital identities are stolen during a cyberattack and sold wholesale on the dark web. Such leaked credentials play a crucial role in open-source intelligence gathering and are frequently exploited in targeted phishing campaigns. However, it has not been clear until now if—and how often—these credentials are used in automated password-guessing attacks on online services. This study attempted to answer that question.

To get closer to the answer, we first procured a large-scale dataset of leaked credentials containing more than 27 billion passwords. We developed a complex data normalization pipeline, which allowed us to efficiently structure and clean these fragmented datasets, yielding over 4 billion unique extracted records and approximately 1.1 billion unique passwords. We believe that this research significantly extends similar efforts in the scientific literature, both in terms of size and recency of the data, and is comparable in size to the state-of-the-art public or commercial databases of closed nature [37].

Afterwards, we conducted an extensive honeypot-based authentication study by collecting data over a one-year period from 39 honeypots across 14 different service types deployed in public, academic, and critical infrastructure networks. This included honeypots in several of the flashpoint regions, such as Ukraine, Russia, and Israel. Special care was taken to identify valid authentication credentials from the requests, taking into account different protocols, authentication mechanisms, and service endpoints. We captured more than 89 million SSH and Telnet events, including nearly 18 million login attempts from almost 423,000 unique IP addresses. Additionally, we captured over 754,000 HTTP requests, nearly 71,000 of which were service login attempts. We extracted over 99,000 unique usernames and nearly 261,000 unique passwords from SSH/Telnet attacks, as well as almost 3500 unique usernames and over 8500 unique passwords from attacks on HTTP services.

The combined dataset allowed us to investigate how, when, and whether leaked credentials are actively used in real-world cyberattacks, providing valuable information on password reuse, credential mutation, attack strategies, and adversarial adaptations to honeypot deception techniques. We analyzed the datasets for domain names and mobile application identifiers, revealing google.com and com.facebook.katana as the most common service domain and application identifier in the leaked data, respectively. We reviewed the password occurrence frequencies and their characteristics, length, and complexity, showing that the prevalent leaked password characteristics included a seven to eight-character-long combination of lowercase letters and digits. We also reviewed the general recorded attack characteristics by analyzing source IP addresses, originating autonomous systems and countries, most commonly targeted honeypot technologies and countries of deployment, as well as attack patterns, and we identified attacker tools and executed commands.

To obtain further insight into both datasets, we extended the analyses using a wide variety of dictionaries, wordlists, and default credential lists commonly used for password cracking, as well as with datasets of generated credentials, using hashcat rules and a heuristic password generator for keywalk patterns. We scanned the datasets for (inter)national phone number patterns, personally identifiable information, and password structures resembling patterns of a particular password-generation software. We analyzed username and password data for code injection attempts and dictionary coverage in three languages, and we assessed the statistical significance of password composition variety between the three major datasets using pairwise chi-square tests on normalized data. Thus, one of the strongest contributions of this study is its multi-faceted approach, incorporating both isolated analyses of massive leaked datasets and honeypot credentials, followed by comparative analyses, cross-referencing the data, and tracking how credentials are used and reused across different systems.

This study provides several critical takeaways for cybersecurity research and practice. The results indicate that commonly used penetration testing password wordlists capture a significant portion of real-world attacks, with some wordlists completely overlapping with the leaked datasets. Attackers also appear to rely more on prebuilt dictionaries than on generative algorithms like keywalks, using such synthetic passwords only as part of the wordlists. Users, however, tend to rely more heavily on such techniques, which implies that the adoption of keywalk detection could present a valuable addition to password strength meters. Another important finding is the discovery of a very limited use of leaked credentials containing email addresses and personally identifiable information in the attacks on our honeynet deployment. Moreover, the presence of either random or long and unique passwords in honeypot data suggests that attackers attempt to detect the honeypots themselves, indicating an adversarial arms race in deception and detection. Finally, the sector of the deployed honeypots (academic, critical, or public cloud) did not present any statistically significant difference between password characteristics, whereas these were noticeable between the attacked protocols (HTTP vs. SSH/Telnet) and leaked data, although affected by large dataset size differences.

However, despite its strengths, the study also faces limitations. Firstly, password comparison was hindered by large amounts of noisy brute-forcing attempts that can quickly drown out smaller, more targeted password-guessing attempts. Secondly, while the honeypot dataset is large and diverse, its coverage of real-world attacker behavior is inherently limited to the services and deployment locations chosen. Almost certainly, many high-value credential leak reuse attempts have not been observed in attacks simply because our honeypots were likely not the primary targets of those attacks, and due to the low profile of the used domain names. While we did deploy highly interactive services into real critical infrastructure networks, these were likely still below the threshold of interest of an advanced persistent threat actor. Thirdly, while we observed some overlap between leaked credentials and honeypot authentication attempts, this overlap was relatively limited, meaning that attackers are not simply reusing leaked credentials en masse but rather adapting their attack strategies dynamically, which could be explained by cost/benefit optimization. Additionally, the absence of real user interactions on honeypots means that some sophisticated attack techniques—such as phishing-based follow-ups or human-driven lateral movement—remain outside the scope of this research. Finally, the complexity of credential leaks and dictionary curation introduces some ambiguity in measuring true attacker intent, particularly when assessing the evolution of password reuse in active attacks.

This research opens several avenues for future exploration. One major direction is expanding honeypot coverage to include more services, protocols, and wider geographic distribution, helping to capture a broader spectrum of real-world credential-based attacks. Additionally, honeytokens could be used in conjunction with honeypots to track the reverse path from honeypots to leaks. To improve the effectiveness of the infrastructure for capturing attacker activity and maximize the probability of detecting leaked credentials, the use of high-profile expired domain names could be explored—such domains could be used to capture latent targeted password-guessing attempts. Additionally, deeper behavioral modeling of attackers—such as tracking attacker persistence, response to failed attempts, and adaptation over time—could help better distinguish automated bot-driven attacks from human-driven credential exploitation. Moreover, behavioral modeling of end users also presents a promising direction for future work, with automated (simulated) user activity raising the perceived value of the honeypots to the attackers. Large language models also appear to be a promising tool with potential uses for improving the adaptive nature of the honeypot operation and user modeling. We are also exploring machine learning and deep learning approaches for further clustering of credentials and broader attack data. Another key area is improving password strength evaluation beyond standard complexity metrics, leveraging machine learning-based pattern recognition to detect evolving attacker strategies. Finally, further adversarial deception research could explore honeypot counter-evasion techniques, focusing on how attackers identify decoy environments and how defenses can evolve to counteract these tactics.

## Figures and Tables

**Table 1:** List of the developed honeypot services, their level of interaction, protocol, and honeypot deployment configurations, including number of instances and target deployment network types.

Honeypot	Instances	Level of Interaction	L7 Protocol	Deployment Network Types
Linux system (SSH)	30	High	SSH	Cloud, Academic, Critical
Linux system (Telnet)	27	High	Telnet	Cloud, Academic, Critical
VMware vCenter Server v6.5	7	Low/Medium	HTTP	Cloud, Academic, Critical
IBM Storwize v7000	3	Low/Medium	HTTP	Cloud, Academic, Critical
QNAP NAS	3	Low/Medium	HTTP	Cloud, Academic, Critical
phpMyAdmin v5.1.1	3	High	HTTP	Cloud, Academic
HP Color LaserJet m552	3	Low/Medium	HTTP	Cloud, Academic
Joomla CMS v3	2	High	HTTP	Cloud, Academic
Joomla CMS v4	2	High	HTTP	Cloud, Academic
OpenStack v17	2	Low/Medium	HTTP	Cloud, Academic
EPSON c20600	2	Low/Medium	HTTP	Cloud, Academic
QSAN NAS	2	Low/Medium	HTTP	Cloud, Academic
Thecus NAS	2	Low/Medium	HTTP	Cloud, Academic
APC SmartUPS	3	Low/Medium	HTTP	Cloud, Critical

**Table 2:** Processed leaked credentials.

Imported Records	Count
Number of imported records	27,608,507,147
Records containing email addresses	26,558,840,633
Records with missing/invalid username or password	402,141,095
Records including service login URLs or application IDs	307,605,875

**Table 3:** Resulting unique leaked data.

Unique Records	Count
Number of unique records	3,987,677,320
Unique usernames	1,509,519,825
Unique passwords	1,096,508,611
Unique emails	2,458,614,185
Unique user and password pairs	2,894,067,919
Unique email and password pairs	3,781,658,942
Unique email domains	27,620,706
Unique service login domains	7,848,100
Unique application IDs	161,120

**Table 4:** Overview of the most common domains (SLD and TLD) and application identifiers in the acquired leaked datasets.

Top 10 Email Domains	Count	Relative (%)
yahoo.com	491,837,626	13
mail.ru	488,695,780	13
hotmail.com	400,261,562	10
gmail.com	391,612,680	10
rambler.ru	208,594,644	5
yandex.ru	178,583,634	5
aol.com	95,290,730	2
bk.ru	89,522,799	2
list.ru	72,945,472	2
inbox.ru	68,856,041	2
**Top 10 Email TLDs**	**Count**	**Relative (%)**
.com	1,739,252,307	45
.ru	1,256,700,095	33
.de	133,991,410	3
.net	125,174,862	3
.fr	98,935,849	3
.uk	73,555,873	2
.it	69,580,480	2
.pl	42,835,630	1
.jp	29,583,847	1
.cz	20,406,405	1
**Top 10 Service Domains**	**Count**	**Relative (%)**
google.com	9,743,448	5
facebook.com	7,848,284	4
live.com	4,059,041	2
roblox.com	3,613,447	2
instagram.com	1,738,736	1
discord.com	1,619,642	1
twitter.com	1,602,900	1
twitch.tv	1,414,635	1
amazon.com	1,346,273	1
netflix.com	1,281,606	1
**Top 10 Service TLDs**	**Count**	**Relative (%)**
.com	110,053,899	61
.br	7,629,400	4
.net	6,859,736	4
.in	3,735,300	2
.org	3,600,508	2
.id	2,210,036	1
.tv	1,888,017	1
.fr	1,790,408	1
.it	1,697,014	1
.vn	1,385,170	1
**Top 10 Application/Bundle IDs**	**Count**	**Relative (%)**
com.facebook.katana	742,150	8
com.instagram.android	565,567	6
com.netflix.mediaclient	457,705	5
com.roblox.client	393,080	4
com.discord	345,751	4
com.spotify.music	330,989	4
tv.twitch.android.app	276,330	3
com.snapchat.android	153,335	2
com.twitter.android	140,184	1
com.facebook.orca	118,603	1

**Table 5:** Most common keywalking patterns in leaked passwords.

Records	Occurrence	Relative (%)
123456	23,420,936	0.93
123456789	13,582,273	0.54
qwerty	10,585,869	0.42
12345	7,507,693	0.30
12345678	4,221,747	0.17
1234567890	2,797,246	0.11
1234567	2,713,098	0.11
qwertyuiop	1,802,821	0.07
1234	1,532,094	0.06
qwert	1,378,993	0.06
654321	1,341,170	0.05
123	1,178,946	0.05
987654321	893,808	0.04
zxcvbnm	890,657	0.04
asdfgh	546,929	0.02

**Table 6:** The most common originating autonomous system and countries attacking HTTP honeypots.

Autonomous System	Requests Originated (%)
DIGITALOCEAN-ASN	10.79
Akamai Connected Cloud	5.57
GOOGLE-CLOUD-PLATFORM	3.16
M247 Europe SRL	2.44
AltusHost B.V.	2.42
CENSYS-ARIN-01	2.38
HURRICANE	2.35
IP Volume inc	2.29
UAB Host Baltic	2.29
Red Byte LLC	2.05
**Source Country**	**Requests Originated (%)**
United States	24.49
Netherlands	7.48
United Kingdom	5.95
Germany	5.88
China	4.51
Russia	4.11
India	3.05
France	2.87
Singapore	2.77
Mexico	2.70

**Table 7:** HTTP authentication requests by honeypot technology.

Honeypot Technology	Valid Authentication Requests ^†^
QNAP NAS	60,309
APC SmartUPS	1481
Thecus NAS	11
Joomla CMS v3	11
EPSON c20600	3
VMware vCenter Server v6.5	2
Joomla CMS v4	2
**Honeypot Technology**	**Invalid Authentication Requests ^†^**
phpMyAdmin v5.1.1	1436
IBM Storwize v7000	1426
QNAP NAS v3.3.6	1265
QSAN NAS	1080
EPSON c20600	1055
HP Color LaserJet m552	1030
APC SmartUPS VT 20 kVA	638
VMware vCenter Server v6.5	506
Joomla CMS v3	140
Thecus NAS	116
OpenStack v17.0.5 Queens	101
Joomla CMS v4	94

^†^ Note that requests targeting the wrong authentication URL for the specific service are not considered login requests for that service (e.g., /boaform/admin/formLogin for services below), even if these are valid login requests for other services. These are not encompassed in the table of per-honeypot-technology statistics.

**Table 8:** Username and password occurrences (all HTTP login requests).

Username	Occurrences
admin	49,896
something	8444
root	258
user	235
support	215
administrator	207
qnap	195
ec8	194
manager	191
adminisp	184
**Password (Decoded)**	**Occurrences**
Feefifofum (Feefifofum)	10,772
cGFzc3dvcmQ= (password)	2038
MTIzNDU2Nzg= (12345678)	1978
MTIzNDU2Nw== (1234567)	1977
MTIzNDU= (12345)	1975
MTIzNDU2Nzg5 (123456789)	1972
bmljb2xl (nicole)	1968
YWJjMTIz (abc123)	1965
aWxvdmV5b3U= (iloveyou)	1960
cHJpbmNlc3M= (princess)	1960

**Table 9:** Username and password occurrences (valid HTTP login requests).

Username	Occurrences
admin	28,710
administrator	200
root	196
qnap	195
support	195
manager	191
jamie	72
kelly	72
tracy	71
jean	71
**Password**	**Occurrences**
password	2038
12345678	1979
1234567	1978
12345	1977
123456789	1973
nicole	1968
abc123	1966
123456	1963
iloveyou	1960
princess	1960

**Table 10:** The most common originating autonomous systems and countries attacking SSH and Telnet honeypots.

Autonomous System	Originating Requests (%)
Chinanet	28.26
DIGITALOCEAN-ASN	8.28
Tencent Building, Kejizhongyi Avenue	4.42
Shenzhen Tencent Computer Systems	3.86
Viettel Corporation	2.67
OVH SAS	2.35
CHINA UNICOM China169 Backbone	1.65
Korea Telecom	1.46
Iran Telecommunication Company PJS	1.42
Cellcom Fixed Line Communication L.P	1.17
**Source Country**	**Originating Requests (%)**
China	39.58
United States	8.93
Vietnam	4.85
Singapore	4.25
India	3.98
Russia	3.19
Germany	2.92
South Korea	2.71
Iran	2.39
Australia	2.06

**Table 11:** Top 10 usernames, passwords, and their combinations during SSH/Telnet login attempts.

Username	Attempt Count
root	12,649,542
admin	946,557
user	230,774
ubuntu	174,173
test	96,962
ubnt	87,207
guest	82,315
debian	75,827
pi	70,617
oracle	65,799
**Password**	**Attempt Count**
123456	557,409
1234	277,427
admin	221,642
123	186,565
password	152,866
root	132,847
1	101,438
12345	98,261
12345678	89,391
admin123	80,314
**Username:Password**	**Attempt Count**
admin:1234	160,193
root:root	104,061
admin:admin	95,241
root:admin	83,386
root:123456	63,922
admin:admin123	56,700
user:user	56,193
root:1234	45,685
root:password	45,086
root:12345	43,358

**Table 12:** Most common usernames during SSH public key authentication attempts.

Username	Attempt Count
root	36,216
admin	12,649
udatabase	2551
ubuntu	202
uftp	188
support	44
aiven	32
0	27
tty0	24
000	15

**Table 13:** Most common SSH/Telnet client versions reported.

SSH Client Version	Percentage (%)
SSH-2.0-Go	44.02
SSH-2.0-libssh_0.9.6	22.88
SSH-2.0-PUTTY	17.94
SSH-2.0-AsyncSSH_2.1.0	4.57
SSH-2.0-libssh2_1.4.3	2.12
SSH-2.0-OpenSSH_7.4	1.48
SSH-2.0-PuTTY	0.86
SSH-2.0-OpenSSH_6.0p1 Debian-4+deb7u2	0.56
SSH-2.0-libssh2_1.9.0	0.54
SSH-2.0-libssh_0.9.5	0.45

**Table 14:** Chi-square test results comparing SSH, HTTP, and Leaks datasets by length and character composition.

Dataset	$\chi^{2}$	*p*-Value	df	α	$C_{V}$
SSH/Telnet vs. HTTP	4120.08	<0.0001	54	0.05	0.0351
SSH/Telnet vs. Leaks	3012.14	<0.0001	54	0.05	0.0018
HTTP vs. Leaks	6379.98	<0.0001	54	0.05	0.0437

**Table 15:** Chi-square test results comparing the password composition between datasets obtained from the critical, academic, and cloud deployment networks of honeypots.

Sectors (HTTP)	$\chi^{2}$	*p*-Value	*df*	α	$C_{V}$
Critical vs. Academic	11.57	0.9897	25	0.05	0.0680
Critical vs. Cloud	14.19	0.9705	26	0.05	0.0739
Academic vs. Cloud	0.77	1.0000	26	0.05	0.0172
**Sectors (SSH/Telnet)**	$\chi^{2}$	***p*** **-value**	***df***	α	$C_{V}$
Critical vs. Academic	2.54	1.0000	43	0.05	0.0243
Critical vs. Cloud	1.73	1.0000	43	0.05	0.0201
Academic vs. Cloud	7.51	1.0000	43	0.05	0.0418

**Table 16:** Coverage and Jaccard indices between the collected username datasets and English dictionaries.

A vs. B	IoU	A Coverage (%)	B Coverage (%)
HTTP vs. OPTED	0.00	0.38	0.01
HTTP ^†^ vs. OPTED		0.02	0.01
HTTP vs. WordNet	0.00	33.75	0.42
HTTP ^†^ vs. WordNet		20.89	9.31
HTTP vs. Wiktionary	0.00	37.95	0.05
HTTP ^†^ vs. Wiktionary		63.60	3.29
SSH/Telnet vs. OPTED	0.00	0.15	0.14
SSH/Telnet ^†^ vs. OPTED		0.34	>100
SSH/Telnet vs. WordNet	0.03	7.45	5.02
SSH/Telnet ^†^ vs. WordNet		79.12	>100
SSH/Telnet vs. Wiktionary	0.01	10.86	0.85
SSH/Telnet ^†^ vs. Wiktionary		86.85	>100
Leaks vs. OPTED	0.00	0.00	41.37
Leaks ^†^ vs. OPTED		0.05	>100
Leaks vs. WordNet	0.00	0.01	52.23
Leaks ^†^ vs. WordNet		1.19	>100
Leaks vs. Wiktionary	0.00	0.03	37.50
Leaks ^†^ vs. Wiktionary		1.66	>100

^†^ Denotes a non-deduplicated dataset used for measuring prevalence. A coverage indicates what percentage of dataset A exists as full-string matches in dataset B, and vice versa for B coverage.

**Table 17:** Coverage and Jaccard indices between the collected password datasets and English dictionaries.

A vs. B	IoU	A Coverage (%)	B Coverage (%)
HTTP vs. OPTED	0.00	0.14	0.01
HTTP ^†^ vs. OPTED		0.05	0.03
HTTP vs. WordNet	0.01	22.88	1.30
HTTP ^†^ vs. WordNet		30.56	12.82
HTTP vs. Wiktionary	0.00	26.69	0.18
HTTP ^†^ vs. Wiktionary		34.65	1.69
SSH/Telnet vs. OPTED	0.00	0.19	0.44
SSH/Telnet ^†^ vs. OPTED		1.62	>100
SSH/Telnet vs. WordNet	0.03	3.86	6.83
SSH/Telnet ^†^ vs. WordNet		16.50	>100
SSH/Telnet vs. Wiktionary	0.01	5.74	1.18
SSH/Telnet ^†^ vs. Wiktionary		20.54	>100
Leaks vs. OPTED	0.00	0.00	37.48
Leaks ^†^ vs. OPTED		0.15	>100
Leaks vs. WordNet	0.00	0.01	50.19
Leaks ^†^ vs. WordNet		3.22	>100
Leaks vs. Wiktionary	0.00	0.04	32.12
Leaks ^†^ vs. Wiktionary		4.37	>100

^†^ Denotes a non-deduplicated dataset used for measuring prevalence. A coverage indicates what percentage of dataset A exists as full-string matches in dataset B, and vice versa for B coverage.

**Table 18:** Per-D-dataset top 10 most common passwords found in the English Wiktionary.

Rank	HTTP Password	SSH/Telnet Password	Leaked Password
1	password	admin	qwerty
2	monkey	password	password
3	princess	root	qwertyuiop
4	daniel	user	dragon
5	babygirl	test	monkey
6	lovely	support	asdfghjkl
7	admin	guest	tinkle
8	qwerty	ubuntu	football
9	master	qwerty	master
10	superman	default	killer

**Table 19:** Coverage and Jaccard indices between the collected username datasets and Russian or German dictionaries.

A vs. B	IoU	A Coverage (%)	B Coverage (%)
HTTP vs. Wiktionary (RU)	0.00	0.00	0.00
SSH/Telnet vs. Wiktionary (RU)	0.00	0.00	0.00
Leaks vs. Wiktionary (RU)	0.00	0.00	0.00
Leaks ^†^ vs. Wiktionary (RU)		0.00	0.39
HTTP vs. Wiktionary (DE)	0.00	5.45	0.01
HTTP ^†^ vs. Wiktionary (DE)		1.09	0.08
SSH/Telnet vs. Wiktionary (DE)	0.00	1.37	0.16
SSH/Telnet ^†^ vs. Wiktionary (DE)		1.49	30.93
Leaks vs. Wiktionary (DE)	0.00	0.01	8.83
Leaks ^†^ vs. Wiktionary (DE)		0.24	>100

^†^ Denotes a non-deduplicated dataset used for measuring prevalence. A coverage indicates what percentage of dataset A exists as full-string matches in dataset B, and vice versa for B coverage.

**Table 20:** Coverage and Jaccard indices between the collected password datasets and Russian or German dictionaries.

A vs. B	IoU	A Coverage (%)	B Coverage (%)
HTTP vs. Wiktionary (RU)	0.00	0.00	0.00
SSH/Telnet vs. Wiktionary (RU)	0.00	0.00	0.00
Leaks vs. Wiktionary (RU)	0.00	0.00	8.70
Leaks ^†^ vs. Wiktionary (RU)		0.03	>100
HTTP vs. Wiktionary (DE)	0.00	2.40	0.02
HTTP ^†^ vs. Wiktionary (DE)		1.05	0.07
SSH/Telnet vs. Wiktionary (DE)	0.00	0.73	0.22
SSH/Telnet ^†^ vs. Wiktionary (DE)		3.54	73.51
Leaks vs. Wiktionary (DE)	0.00	0.01	8.60
Leaks ^†^ vs. Wiktionary (DE)		0.48	>100

^†^ Denotes a non-deduplicated dataset used for measuring prevalence. A coverage indicates what percentage of dataset A exists as full-string matches in dataset B, and vice versa for B coverage.

**Table 21:** Coverage and Jaccard indices between the collected password datasets and various wordlists.

A vs. B	IoU	A Coverage (%)	B Coverage (%)
HTTP vs. FastTrack	0.01	0.70	22.52
HTTP vs. John	0.22	25.40	60.20
HTTP vs. Nmap	0.57	58.06	97.56
HTTP vs. RockYou	0.00	79.30	0.05
HTTP vs. Wifite	0.01	20.10	0.83
SSH/Telnet vs. FastTrack	0.00	0.05	48.85
SSH/Telnet vs. John	0.01	1.24	90.97
SSH/Telnet vs. Nmap	0.02	1.70	88.58
SSH/Telnet vs. RockYou	0.01	28.60	0.52
SSH/Telnet vs. Wifite	0.04	6.11	7.83
Leaks vs. FastTrack	0.00	0.00	96.56
Leaks vs. John	0.00	0.00	100.00
Leaks vs. Nmap	0.00	0.00	100.00
Leaks vs. RockYou	0.01	0.81	61.98
Leaks vs. Wifite	0.00	0.02	82.01

**Table 22:** Coverage and Jaccard indices between the username and password pairs from the collected credential datasets and various default account lists.

A vs. B	IoU	A Coverage (%)	B Coverage (%)
HTTP vs. Nessus	0.00	0.12	19.08
HTTP vs. DPL4Hydra	0.00	0.32	1.84
HTTP vs. Mirai	0.00	0.09	36.67
HTTP vs. Piata SSH	0.01	0.88	24.44
HTTP vs. Routers	0.00	0.22	11.59
SSH/Telnet vs. Nessus	0.00	0.03	82.24
SSH/Telnet vs. DPL4Hydra	0.00	0.17	16.80
SSH/Telnet vs. Mirai	0.00	0.01	100.00
SSH/Telnet vs. Piata SSH	0.00	0.20	97.43
SSH/Telnet vs. Routers	0.00	0.10	91.72
Leaks vs. Nessus	0.00	0.00	50.00
Leaks vs. DPL4Hydra	0.00	0.00	14.37
Leaks vs. Mirai	0.00	0.00	71.67
Leaks vs. Piata SSH	0.00	0.00	70.54
Leaks vs. Routers	0.00	0.00	50.72

**Table 23:** Coverage and Jaccard indices between unique usernames, passwords, and credential pairs from collected datasets.

A vs. B	IoU	A Coverage (%)	B Coverage (%)
HTTP vs. SSH/Telnet (Usernames)	0.02	90.35	1.67
HTTP vs. Leaks (Usernames)	0.00	95.97	0.00
SSH/Telnet vs. Leaks (Usernames)	0.00	83.45	0.01
HTTP vs. SSH/Telnet (Passwords)	0.02	72.52	2.33
HTTP vs. Leaks (Passwords)	0.00	91.27	0.00
SSH/Telnet vs. Lekas (Passwords)	0.00	67.40	0.02
HTTP vs. SSH/Telnet (Cred. Pairs)	0.01	16.18	0.94
HTTP vs. Leaks (Cred. Pairs)	0.00	65.91	0.00
SSH/Telnet vs. Leaks (Cred. Pairs)	0.00	25.24	0.00

**Table 24:** Top 10 keywalk passwords in the HTTP dataset.

Password	Occurrence	Relative (%)
12345678	1979	3.1993
1234567	1978	3.1977
12345	1977	3.1960
123456789	1973	3.1896
123456	1963	3.1734
654321	996	1.6101
1234	50	0.0808
1234567890	40	0.0646
qwerty	38	0.0614
qwertyuiop	35	0.0565

**Table 25:** Incidence of password mutation across HTTP, SSH/Telnet, and leaked datasets. The table presents normalized edit distances (Levenshtein-based) and their distribution, with the most common mutated password in each bin.

Dataset	Mutation Bin	Percentage (%)	Top Password
HTTP	0.0	3.29	password
HTTP	0.1	0.12	password1
HTTP	0.2	0.07	Password1
HTTP	0.3	0.02	password123
SSH/Telnet	0.0	0.84	password
SSH/Telnet	0.1	0.25	passw0rd
SSH/Telnet	0.2	0.52	p@ssw0rd
SSH/Telnet	0.3	0.33	password123
Leaks	0.0	0.21	password
Leaks	0.1	0.07	password1
Leaks	0.2	0.05	paSSword
Leaks	0.3	0.02	password123
HTTP	0.0	3.17	monkey
HTTP	0.1	0.04	monkey1
HTTP	0.2	0.04	donkey
HTTP	0.3	0.12	hockey
SSH/Telnet	0.0	0.01	monkey
SSH/Telnet	0.1	0.01	monkey!
SSH/Telnet	0.2	0.02	money
SSH/Telnet	0.3	0.04	mickey
Leaks	0.0	1.91	monkey
Leaks	0.1	0.88	monkey1
Leaks	0.2	1.57	money
Leaks	0.3	3.36	money1
HTTP	0.0	0.05	killer
HTTP	0.1	0.01	killer1
HTTP	0.2	0.01	miller
HTTP	0.3	0.05	silver
SSH/Telnet	0.0	0.01	killer
SSH/Telnet	0.1	0.00	killer7
SSH/Telnet	0.2	0.01	miller
SSH/Telnet	0.3	0.03	silver
Leaks	0.0	0.87	killer
Leaks	0.1	0.49	killer1
Leaks	0.2	1.15	miller
Leaks	0.3	2.90	silver

## Data Availability

The datasets presented in this article are not readily available due to their sensitive nature and privacy restrictions. Special requests to access the datasets in part should be directed to the corresponding author.
